# Prophylactic and therapeutic vaccination protects sperm health from *Chlamydia muridarum*-induced abnormalities

**DOI:** 10.1093/biolre/ioad021

**Published:** 2023-02-17

**Authors:** Emily R Bryan, Logan K Trim, Pawel Sadowski, Selvam Paramasivan, Jay J Kim, Kyle Gough, Sophia Worley, Toby I Maidment, Alison J Carey, Bettina Mihalas, Eileen A McLaughlin, Kenneth W Beagley

**Affiliations:** Queensland University of Technology, School of Biomedical Sciences and Centre for Immunology and Infection Control, Faculty of Health, Herston, QLD, Australia; Queensland University of Technology, School of Biomedical Sciences and Centre for Immunology and Infection Control, Faculty of Health, Herston, QLD, Australia; Queensland University of Technology, Central Analytical Research Facility, Brisbane, QLD, Australia; Queensland University of Technology, Central Analytical Research Facility, Brisbane, QLD, Australia; Queensland University of Technology, School of Biomedical Sciences and Centre for Immunology and Infection Control, Faculty of Health, Herston, QLD, Australia; Queensland University of Technology, School of Biomedical Sciences and Centre for Immunology and Infection Control, Faculty of Health, Herston, QLD, Australia; Queensland University of Technology, School of Biomedical Sciences and Centre for Immunology and Infection Control, Faculty of Health, Herston, QLD, Australia; Queensland University of Technology, School of Biomedical Sciences and Centre for Immunology and Infection Control, Faculty of Health, Herston, QLD, Australia; Queensland University of Technology, School of Biomedical Sciences and Centre for Immunology and Infection Control, Faculty of Health, Herston, QLD, Australia; School of Environmental and Life Sciences, Faculty of Science, The University of Newcastle, University Drive, Callaghan, NSW, Australia; School of Environmental and Life Sciences, Faculty of Science, The University of Newcastle, University Drive, Callaghan, NSW, Australia; Faculty of Science, Medicine & Health, University of Wollongong, Keiraville, NSW, Australia; Faculty of Science, University of Auckland, Auckland, New Zealand; Queensland University of Technology, School of Biomedical Sciences and Centre for Immunology and Infection Control, Faculty of Health, Herston, QLD, Australia

**Keywords:** *Chlamydia*, male infertility, prophylactic vaccine, therapeutic vaccine, sperm, testis

## Abstract

*Chlamydia* is the most common bacterial sexually transmitted infection worldwide and it is widely acknowledged that controlling the rampant community transmission of this infection requires vaccine development. In this study, for the first time, we elucidate the long-term response to male mouse chlamydial vaccination with chlamydial major outer membrane protein (MOMP) and ISCOMATRIX (IMX) both prophylactically and in a novel therapeutic setting. Vaccination significantly reduced and, in some cases, cleared chlamydial burden from the prostates, epididymides, and testes, which correlates with high IgG and IgA tires in tissues and serum. Important markers of sperm health and fertility were protected including sperm motility and proteins associated with fertility in men. Within splenocytes, expression of IFNγ, TNFα, IL17, IL13, IL10, and TGFβ were changed by both infection and vaccination within CD4 and CD8 T cells and regulatory T cells. Within the testicular tissue, phenotypic and concentration changes were observed in macrophages and T cells (resident and transitory). This revealed some pathogenic phenotypes associated with infection and critically that vaccination allows maintenance of testicular homeostasis, likely by preventing significant influx of CD4 T cells and promoting IL10 production. Finally, we demonstrated the testes contained immature (B220^+^) B cells and mature (CD138^+^) *Chlamydia*-specific plasma cells. Thus, through vaccination, we can maintain the healthy function of the testes, which is vital to protection of male fertility.

## Teaser

Pro-inflammatory and regulatory T cells and B cells secreting IgG and IgA are elicited by prophylactic and therapeutic vaccination to reduce male reproductive tract chlamydial burden, protecting the testicular environment and spermatogenesis from damage normally induced by chlamydial infection.

## Graphical abstract

**Figure:**
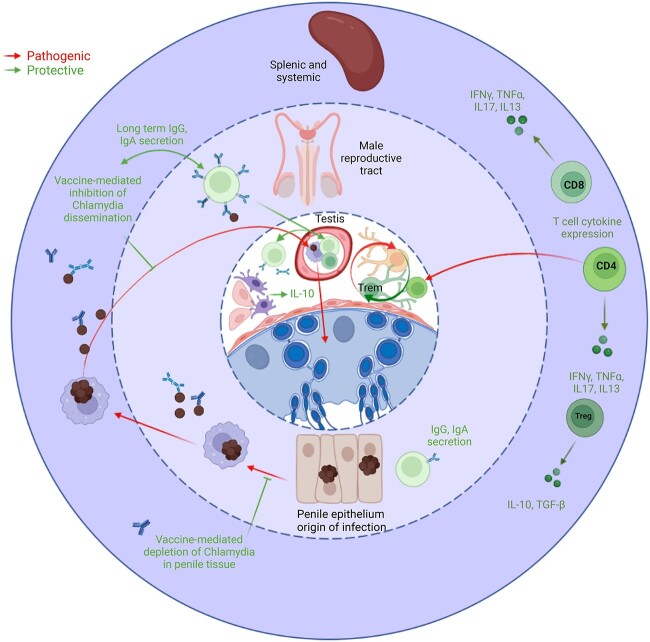

## Introduction

The impact of the sexually transmitted bacterial pathogen *Chlamydia* on reproductive health is being established and characterized more thoroughly now than ever before, although the focus remains on females. There are approximately 127 million genital *Chlamydia* infections globally each year [1], and in many countries where screening of both men and women is routinely available, almost half of reported infections are in males. Chlamydial surveillance is likely dramatically underestimated though, owing to the predominantly asymptomatic nature of infections; 60–80% in men and women [2, 3]. As such, the potential of this common sexually transmitted infection (STI) to form an unrecognized reservoir in the community and impact the reproductive potential of the population is immense. Already, around 15% of male infertility may be due to STIs like *Chlamydia*, a large contribution to male infertility, which accounts for 40–50% of all infertility cases [4].

In males (humans and animals), infection initially infects the penile urethra and then disseminates throughout the urogenital and reproductive tracts [5–9]. *Chlamydia muridarum* infection of mouse reproductive tracts closely reflects what occurs in human female and male infections (although less is known about male infections). The model is well accepted as a research tool with one weakness: that transmission cannot be effectively studied as females can only be infected in diestrus. *Chlamydia* likely contributes to male subfertility by causing inflammatory damage to the male reproductive tract including the urethra, prostate, epididymides, and testes [3]. The testes are vital for male fertility, as the site of sperm production. *Chlamydia* infects and actively replicates in the testes of humans and other mammalian species [5, 7, 10, 11], although infection-induced damage to the testicular environment has predominantly been characterized in animal models. Damage includes DNA fragmentation and death of key testicular cells (Sertoli and germ cells) [6, 12, 13]. This likely causes breakdown of the blood-testis barrier, formed by tight junctions between neighboring Sertoli cells within the testis seminiferous tubule epithelium, which dysregulates the immune privilege and suppression present under normal conditions [10].

The loss of the blood-testis barrier and immune privilege will dysregulate spermatogenesis including by excessive exposure of sequestered auto-immunogenic sperm [14]. This is a likely cause of abnormalities present in sperm produced by infected males. In men, *Chlamydia* induces sperm DNA fragmentation, apoptosis, precocious capacitation, abnormal morphology, and low motility [8, 12, 15–18]. In mice, in addition to those same defects, infection reduces oocyte-binding capability and offspring viability, as well as altering offspring development [10]. With the historical neglect of male fertility and reproductive health, the impact of STIs on male health and their offspring is more important and concerning than ever.

Treatment of *Chlamydia* is complex due to factors including non-compliance, antibiotic resistance, and recrudescence, which are some of the major concerns facing conventional use of antibiotics. An additional concern around antibiotic-mediated clearance of infection is the induction of arrested immunity to natural infection, leaving the host more vulnerable to repeated infections [19]. Instead, prevention and treatment of *Chlamydia* infections needs to provide long-term protection against infection and subsequent subfertility, for which modeling has repeatedly shown vaccination is the most efficient method to achieve this [20, 21]. Multiple vaccines have been trialed in animal models, and more recently a vaccine candidate moved into clinical trials in women [22]. Vaccine trials, including this human trial, inclusive of male infections are scarce. A mouse model has shown however, that immunity preventing transmission was only achieved when both males and females are vaccinated, which indicates the need to include males in chlamydial vaccine research [23]. However, the protective effects of vaccination on male fertility were not evaluated during these vaccine trials.

Vaccine composition, both antigen and adjuvant, varies widely between trials, introducing comparative difficulties. One commonly used chlamydial antigen is the major outer membrane protein (MOMP), due to its immunogenicity and relative abundance on chlamydial cells [23–26]. Similarly, ISCOMATRIX (IMX) is a well-characterized vaccine adjuvant consisting of saponin, phospholipids, and cholesterol, which is FDA-approved for use in cancer therapeutics [27]. Here, male mice were prophylactically and therapeutically vaccinated with MOMP and IMX with intra-penile challenge by *C. muridarum*, the mouse specific strain of *Chlamydia,* mimicking the natural sexual transmission route. Next, sperm health as a marker of fertility and immunological mechanisms for vaccine-mediated protection were investigated.

## Materials and methods

### Mice

C57BL/6JArc male mice were purchased at 6-weeks-old (Animal Resource Centre, Canning Vale, Western Australia). In accordance with Australian code of practice for the care and use of animals for scientific purposes (National Health and Medical Research Council), mice were housed under PC2 conditions and provided with food and water *ad libitum*. The following experiments were approved by Queensland University of Technology (UAEC #1800001203), the University of Newcastle (UAEC #A-2019-908), and Queensland Institute of Medical Research Berghofer Medical Research Institute (QIMR-B AEC # A1802-600M).

### In vivo *Chlamydia* infection

Mice were infected *via* the intra-penile route, as described previously [23, 28]. Briefly, mice were anaesthetized with 10 mg/kg xylazine (Bayer, North Sydney, New South Wales) and 100 mg/kg ketamine (Parnell Laboratories, Alexandria, New South Wales) and then infected with 1 × 10^6^ inclusion forming units (IFU) of *C. muridarum* Weiss (kindly provided by Dr. Catherine O’Connell, University of North Carolina). In the prophylactic study, the infected groups were infected 2 weeks after the last vaccination. In the therapeutic study, mice were infected 2 weeks before the first vaccination. The non-infected control groups were mock-infected with chlamydial sucrose-phosphate storage buffer (74.6 g/L sucrose, 0.512 g/L KH_2_PO_4_, 1.237 g/L K_2_HPO_4_, pH 7.2).

### Vaccination

Mice were lightly anaesthetized (isoflurane, Zoetis Australia, Rhodes, New South Wales), then 5 μL of solution was administered to each nasal nares for 10 μL in total. The naïve group received sterile PBS, the adjuvant only groups received 10-μg IMX (provided by Zoetis Australia) in PBS, and the vaccinated groups received 50 μg of recombinant *C. muridarum* MOMP [29] and 10-μg IMX in PBS per dose. Four doses in total were given: the first three doses were separated by 7 days, and the fourth 14 days after the third. There were ten mice per group (*n* = 10). The experimental groups included the following:

1. Given PBS only (**naïve**), prophylactic and therapeutic groups.
2. Given IMX alone, then infected (**adjuvant-only**), prophylactic group.
3. Given MOMP+IMX, then infected (**vaccinated**), prophylactic group.
4. Given MOMP+IMX, non-infected (**non-infected-vaccinated**), therapeutic group.
5. Infected, then given IMX alone (**adjuvant-only**), therapeutic group.
6. Infected, then given MOMP+IMX (**vaccinated**), therapeutic group.

**Figure 1:**
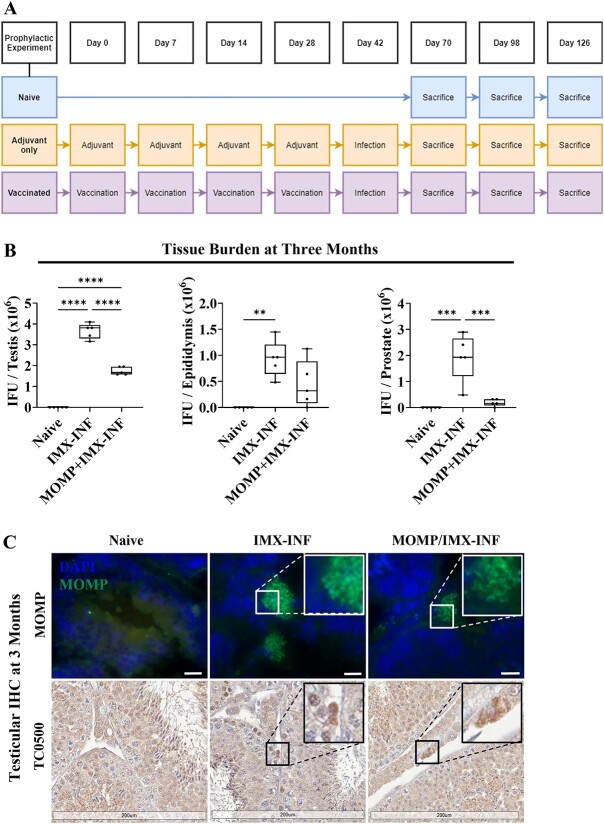
Chlamydial burden in male reproductive tissues. Mice were immunized with ISCOMATRIX (IMX) or MOMP+IMX then infected (INF) with *C. muridarum via* the intra-penile route and sacrificed (A). After 3 months, *C. muridarum* was found in (B) the testes, epididymis, and prostates by culture of live infection from tissue homogenates and (C) the testes by immunohistochemistry (IHC) to confirm the culture results. The IHC included MOMP (green) counterstained with DAPI (blue) where the scale bar = 10 μm and representative images were captured on X100 magnification (Axio Vert.A1, Zeiss), and active replication marker TC0500 (DAB, brown) counterstained with hematoxylin (purple) where the scale bar = 200 μm and slides were scanned (Aperio AT Turbo, Leica) and visualized (Aperio ImageScope v12.4.3.5008). Graphs and statistical analyses (two-way ANOVA with Tukey’s multiple comparisons test, ^*^^*^P < 0.01, ^*^^*^^*^^*^P < 0.0001) were generated in GraphPad Prism (v9.2.0), data are mean ± SD.

Figures 1 and 5 contain detailed experimental timelines.

### Analysis timepoints

Sperm, serum, reproductive tract tissues (testis, epididymis, prostate, penis), and spleens were harvested from mice after their last procedure, i.e. for prophylactically vaccinated mice, samples were harvested 1, 2, and 3 months post-challenge, and for therapeutically vaccinated mice, samples were harvested 1, 3, and 6 months after the last vaccination. Mice were euthanized by cervical dislocation after being deeply anesthetized with isoflurane.

### Sperm analysis

Sperm were isolated from the vas deferens as previously described [6] into Biggers, Whitten, and Whittingham (BWW) media [30] at 290–310 mOsm (Fisk 210 Osmometer, John Morris Group, Sydney, New South Wales). Sperm were incubated for 20 min in BWW media at 37°C, 5% CO_2._ Immediately following swim-up, 1 × 10^6^ sperm were moved to capacitating BWW media and incubated for 20 min, 37°C, 5% CO_2._ Capacitated sperm were assessed in a hemocytometer to quantify the number of progressively motile sperm per milliliter. Capacitated sperm were also incubated in droplets of BWW with C57BL/6JArc mouse oocytes, collected as previously described [31], for 10 min. The sperm-oocyte aggregates were washed to dislodge unbound sperm, then the remainder were viewed using light microscopy to quantify the number of bound sperm for the zona pellucida binding assay [32].

Sperm were also heated and methanol fixed onto glass microscope slides, stained with eosin Y and methylene blue to assess morphology. Sperm were also set into low-melting point agarose for the sperm chromatin dispersion assay (SCDA, as per manufacturer’s instructions, Halomax, Halotech, Madrid, Spain), briefly slides were placed in lysis buffer for five min, washed in reverse osmosis water for 5 min, fixed in 80% v/v ethanol for 2 min and 100% v/v ethanol for 2 min, then stained with SYBR Safe DNA Gel Stain (S33102, Invitrogen, Brisbane, Queensland) mixed with VECTASHIELD mounting medium (Vector Laboratories, Newark, California) (1:1 v/v). Stained sperm were assessed for DNA fragmentation using epifluorescent microscopy [33].

### Chlamydial culture

*Chlamydia muridarum* for mouse infection was grown and purified by established protocol [34]. *Chlamydia muridarum* was cultured from mouse tissues by homogenization for 30 s using a tissue homogenizer with a blade attachment (TH220, OMNI International, Kennesaw, Georgia) into 500 μL of sucrose phosphate glutamine (SPG, 74.6 g/L sucrose, 0.512 g/L KH_2_PO_4_, 1.237 g/L K_2_HPO_4_, 5 mM L-glutamine) and quantified by infection of McCoy cell monolayers (ATCC Cat: CRL-1696) grown in complete RPMI 1640 (10% heat-inactivated fetal calf serum, 2 μg/mL gentamycin, and 100 μg/mL streptomycin sulfate, 1× Glutamax (Thermo Fisher Scientific, Brisbane, Queensland) at 37^o^C, 5% CO_2,_ for 24 h. Monolayers were fixed with 100% v/v methanol, washed twice with PBS and blocked for 1 hour with 5% v/v heat-inactivated fetal calf serum in PBS. Monolayers were incubated with primary sheep anti-MOMP sera (1:500 v/v, [26]) diluted in blocking solution for 1 hour, washed twice with PBST, and incubated for 1 hour with a secondary donkey anti-sheep IgG conjugated to AlexaFluor 488 (2 μg/mL, Thermo Fisher Scientific). Images were captured with epifluorescent microscopy (Zeiss Axio Vert.A1, Carl Zeiss AG, Jena, Germany) and inclusions were quantified.

### Tissue histology and immunohistochemistry (IHC)

Testes were harvested into Bouin’s solution, fixed for 24 h and then washed in 80% v/v ethanol, or alternatively frozen in Tissue Tek O.C.T Compound (Sakura Finetek, Torrance, California). Bouin’s-fixed tissues were processed and paraffin embedded, and both fixed and frozen testes were sectioned (4 μm) onto hydrophilic, poly-D-lysine coated glass microscopy slides (Thermo Fisher Scientific). Fresh tissues were fixed in 100% v/v acetone, endogenous peroxidase was quenched with 2% H_2_O_2_, non-specific binding was blocked with 5% v/v FCS in PBS, and then IHC was competed for chlamydial MOMP as described above.

Fixed tissues underwent processing (Leica ST5010-CV5030 Integrated Workstation), peroxidase quenching, antigen retrieval (Diva Decloaker, Biocare Medical, Pacheco, California), blocking (2% BSA in Background Sniper, Biocare Medical), and then staining. Chlamydial TC0500 staining was performed using primary sheep anti-TC0500 IgG (Recombinant TC0500 antigen produced by Dr Charles Armitage, 1:1000) and secondary donkey anti-sheep-IgG IgG (1:1000, Life Technologies, Brisbane, Queensland) as previously described [7]. Immature B cell staining was performed by QIMR-B core histology facility using primary rat anti-CD45R (B220, Invitrogen) and secondary Rat Probe then Rat Polymer HRP (Biocare Medical), DAB development, before finally being counterstained with hematoxylin. Sections were stained for plasma cells by dewaxing and rehydrating (Leica Autostainer XL2), antigen retrieval in sodium citrate buffer (10mM sodium citrate, 0.05% v/v Tween20, pH 6.0) by microwaving for 20 min while boiling, resting in water at room temperature for 30 min, peroxidase quenching and blocking as above, then probing with anti-mouse CD138 (1:100, 142502, BioLegend, San Diego, California) with secondary (anti-Rat IgG, 1:1000, Thermo Fisher Scientific) or biotinylated MOMP with tertiary SA-HRP (1:500, Thermo Fisher Scientific), and finally DAB development (10 min, room temperature, Sigma Aldrich, Sydney, Australia), and counterstaining with nuclear fast red.

### MOMP production and biotinylation

Recombinant MOMP was produced as previously described [26]. Recombinant MOMP was used for vaccination and biotinylated for use in IHC. Biotinylated-Lysinyl-benzoylphenylalanine was constructed using standard microwave assisted solid phase peptide synthesis techniques on 2-chlorotrityl resin. Chain elongation was affected by the addition of HCTU activated amino acids and FMOC removal by treatment with 30% piperidine. Briefly, 75 mg of resin (1.55 mmol/g loading) were derivatized with 0.01 mmol of Fmoc-4-benzoyl-L-phenylalanine activated with 1.1 Meq of HCTU with microwave assistance in a CEM Discover microwave synthesis system running at 70^o^C and 20 W for 5 min. Deprotection was carried out under similar conditions for 3 min and was followed by the addition of Fmoc-lysine at a four-fold molar excess. Following lysine deprotection, Biotin was added in eight-fold excess, again activated by HCTU but derivatization was extended over a period of 20 min. Completion of biotinylation was monitored using the Kaiser test. The completed peptide was cleaved from the resin and lysine protecting group removed by treatment with 97.5% TFA 2.5% H_2_O (silanes and thiol scavengers were omitted to protect the benzoyl phenylalanine). Peptide was recovered by trituration in 10 volumes of ice-cold methanol. Precipitated peptide was then redissolved in 30% acetonitrile, 70% H_2_O and lyophilized.

Lyophilized peptide was solubilized in anhydrous DMSO to a final concentration of 1 mg/mL. 50 μl of this stock solution was mixed with 50 μl of purified MBP-MOMP (4.8 mg/ml) and incubated on ice for 30 min in the dark. The mixture was then spotted onto parafilm and frozen at −80^°^C for 3 h while supported by a steel tube block. The spotted material and steel block were then placed in a UV cross-linking chamber (Uvitec) and irradiated with a dose of 1000 J of UVA radiation. 100 ng of cross-linked MOMP and a similar amount of unlabeled protein was subject to SDS PAGE and western transfer onto nitrocellulose. Probing with HRP-conjugated streptavidin and ECL detection produced a strong signal for labeled MOMP but no signal for unlabeled protein. The biotinylated MOMP was generously constructed and provided by Prof Jon Harris (QUT).

### Enzyme Linked Immunosorbent Assay

Whole blood was collected *via* cardiac bleed, clotted, then serum collected post-centrifugation. Enzyme Linked Immunosorbent Assay (ELISA) was used to determine the concentration of anti-chlamydial IgG and IgA as previously described [35]. The detection antibodies were for IgG (1:1000, Catalog number 616520, Thermo Fisher Scientific) and for IgA (1:1000, Catalog number 626720, Thermo Fisher Scientific). ELISA was also used to determine the concentration of testosterone in serum of non-infected and infected animals at 6 months post-infection as previously described by Dr Tamara Keeley (University of Queensland, Australia) [36].

### Flow cytometry

#### Splenocytes

Splenocytes were isolated as previously described [26], seeded into 96-well U-bottom microtiter plates, stained with CFSE (C34554, Thermo Fisher Scientific) as per the manufacturer’s instructions, stimulated as previous described [23], and incubated for 72 h (5% CO_2_, 37°C), the last 12 h of which was in the presence of Brefeldin A (00-4506-51, Thermo Fisher Scientific) used as per the manufacturer’s instructions. The single cell suspension was then stained for flow cytometry. Cells were blocked in 5% v/v FCS in PBS for 1 hour, washed into PBS for viability staining (LIVE/DEAD Fixable Aqua Dead Cell Stain Kit, L34966, Thermo Fisher Scientific) as per the manufacturer’s instructions, washed into PBS, then resuspended in the antibody cocktail as per Supplementary Table 1. Cells were then washed twice in PBS (500 g, 5 min per centrifugation), fixed in 1% paraformaldehyde (20 min), washed in PBS, then assayed *via* the flow cytometer (Beckman Coulter Cytoflex S).

#### Testicular interstitial cells

Testicular interstitial cells were isolated as previously described [6]. The single cell suspension was then stained for flow cytometry as above using the antibodies described in Supplementary Table 1, then assayed *via* the flow cytometer (Beckman Coulter Cytoflex S).

### Proteomics

#### Protein extraction and digestion

Sperm samples were mixed with the 50-μL SDC lysis buffer (1 % sodium deoxycholate, 100-mM dithiothreitol, 100-mM Tris-HCl, pH 7.6) and sonicated (5 min). The lysate was centrifuged (16,639 rpm, 5 min, 4 °C) and the supernatant subjected to a modified filter-aided sample preparation [37] in which multiplexed sample preparation was enabled using Pall AcroPrep Advance 30 kDa MWCO Filter Plate (Sigma Aldrich). All buffer exchange steps were conducted using vacuum manifold (8 min) and fully automated on JANUS G3 workstation (PerkinElmer, Glen Waverley, Victoria). Digestion involved addition of 0.1-μg Sequencing Grade Modified Trypsin (Promega, Sydney, New South Wales) and was conducted for 16 h at 37°C. After digestion, peptide samples were acidified by mixing with 4% TFA at 1:1 ratio (v/v).

#### Desalting

Acidified peptide samples were loaded onto activated Empore SCX membrane disk (3M) and subjected to a desalting protocol [38]. The protocol was adapted for 96 format using custom made StageTip support. Like for digestion, all buffer exchange steps were automated and relied on vacuum. Desalted peptides were dried on Ultravap (Porvair Sciences, Wrexham, United Kingdom) and resuspended in a buffer (2% ACN, 0.1% FA) containing iRT peptides (Biognosysis, Schlieren, Switzerland). A pooled biological QC sample (PBQC) was created by combining 1 μL aliquot taken from all desalted samples.

#### Liquid chromatography-tandem mass spectrometry (LC-MS/MS)

All peptide samples were analyzed on TripleTOF 6600 mass spectrometer (SCIEX, Framingham, Massachusetts) coupled to Eksigent ekspert nanoLC 400 liquid chromatograph (Eksigent Technologies, Redwood City, California) and configured for microflow LC applications as described in [39]. Chromatography method utilized ramping mobile phase B from 3 to 25% over 98 min, and then from 25 to 35% over 5 min. After this, the column was washed for 5 min using 80% mobile phase B and re-equilibrated for 8 min using 97% mobile phase A before injecting the next sample. Mass spectrometry method relied on sequential window acquisition of all theoretical mass spectra (SWATH) variable window acquisition strategy as follows. A high resolution (30 000) time of flight (TOF) mass spectrometry (MS) scan was collected over a range of *m/z* 350–1500 for 0.05 s, then a set of 60 overlapping *m/z* ranges (SWATH windows) over the range *m/z* 399.5-1000 were sequentially subjected for high sensitivity TOF MS/MS scans conducted over the range of *m/z* 100–1800 and 0.05 s per window, resulting in the total duty cycle of 3.1 s. For QC purposes, PBQC sample was analyzed three times, at the beginning, in the middle, and at the end of batch. For spectral library generation, an additional PBQC sample analysis was conducted using a mass spectrometry method that utilized data-dependent acquisition (DDA) strategy, In this case, a high-resolution (30 000) TOF MS scan was collected over a range of *m/z* 400–1100 for 0.25 s, followed by a high sensitivity TOF MS/MS scan over the range of *m/z* 100–1500 on up to 40 most abundant peptide ions (0.05 s per spectrum) that had intensity greater than 150 cps and charge state of 2–5. The dynamic exclusion duration was set at 20 s.

#### D‌DA data analysis

Mass spectrometry data file corresponding to DDA run was analyzed using Paragon algorithm [40] embedded in ProteinPilot software (v. 5.0) with the following parameters: identification (sample type), iodoacetamide (cysteine alkylation), trypsin (digestion), urea denaturation (special factors), thorough (search effort). FASTA database was created by merging UniProt Mus musculus protein sequences (downloaded April 2020) with GPM cRAP protein sequences (downloaded May 2018) and Biognosys iRT protein sequence. The result file filtered at 1% FDR was then imported into Skyline software [41] to create a spectral library with Biognosys-11 (iRT-C18) selected as iRT standard peptides for retention time (RT) calibration.

#### SWATH data analysis

Targeted extraction of mass spectrometry data files corresponding to SWATH runs was conducted in Skyline software following published methodology [42], detailed in Supplementary Table 2. The above-mentioned FASTA database was compiled as background proteome and used to associate with relevant proteins a non-redundant list of peptide targets imported from assay library. For RT calibration and prediction, an iRT calculator was created using 11 standard peptides and signals. Peak picking relied on mProphet model [43] built using target-decoy strategy. An FDR of 1% (q value cut-off 0.01) was applied and the peptides passing the cutoff in all replicate sample were kept, resulting in the refined document containing quantitative information for 21 944 peptides corresponding to 2652 proteins. Finally, Skyline report module was used to export fragment-, peptide-, and protein-level data, and used as input to MSstats software [44] where log transformation and global median normalization were applied to data before generating relevant QC and volcano plots.

### Statistical analysis

Sperm parameters, anti-chlamydial antibodies, immune cell subset abundance, and chlamydial burden were analyzed using GraphPad Prism (version 9) software. According to the binomial approximation, to compare the differences between each of the groups, and at the three different timepoints, two-way ANOVA with Tukey’s multiple comparisons test was performed. The level of statistical significance for all tests was set at *P* ≤ 0.05 (^*^P < 0.05, ^*^^*^P < 0.01, ^*^^*^^*^P < 0.001, ^*^^*^^*^^*^P < 0.0001).

## Results

### Prophylactic vaccination reduces reproductive tissue *Chlamydia* burden

The prophylactic vaccination schedule and experimental groups are outlined in Figure 1(A). Tissues within the male reproductive tract were homogenized and viable *Chlamydia* was cultured. Prophylactic vaccination reduced the number of *Chlamydia* IFU per testis at 3 months post-infection (adjuvant-only average = 3.665 × 10^6^ IFU; vaccinated average = 1.749 × 10^6^; *P* < 0.0001, 52% decrease), per epididymis (adjuvant-only average = 0.9324 × 10^6^ IFU; vaccinated average = 0.4501 × 10^6^; NS, 51% decrease), and per prostate (adjuvant-only average = 1.929 × 10^6^ IFU; vaccinated average = 0.1929 × 10^6^; *P* < 0.001, 90% decrease), including one epididymis and prostate that was clear of infection (Figure 1B). No *Chlamydia* was detected in naïve tissues. Inclusions within testes, where the highest burden was present, were confirmed with IHC (Figure 1C) of chlamydial MOMP and inclusions also stained positively for chlamydial TC0500, indicating actively replicating infection.

### Prophylactic vaccination protects or partially protects sperm health

**Figure 2:**
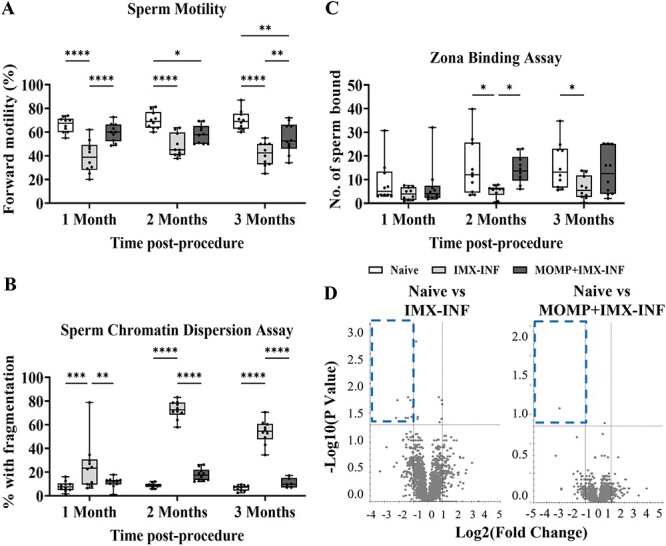
Sperm motility, chromatin dispersion, oocyte-binding, and proteome during *C. muridarum* infection and prophylactic vaccination. Immunization with ISCOMATRIX (IMX) alone or MOMP mixed with IMX was followed by intra-penile *C. muridarum* infection (INF) (or mock with SPG), then after 1, 2, and 3 months, sperm were harvested from the vas deferens of all mice (*n* = 10/group). Sperm were imaged using brightfield microscopy (Axio Ver.A1, Zeiss). (A) There were differences in sperm motility between groups at all timepoints. (B) There were differences in sperm chromatin dispersion at all timepoints. (C) There were differences in oocyte-binding at 2 and 3 months. (D) Sperm were also enzymatically digested, used for mass spectrometry, and analyzed using proteomics bioinformatics. Identified proteins are represented by dots on the volcano plot, with significance thresholds indicated by vertical and horizontal lines, and significantly downregulated proteins highlighted by the blue dashed box. Graphs and statistical analyses (two-way ANOVA with Tukey’s multiple comparisons test, ^*^P < 0.05, ^*^^*^P < 0.01, ^*^^*^^*^^*^P < 0.0001) were generated in GraphPad Prism (v9.2.0), data are mean ± SD.

To assess the impact of chlamydial burden on the testicular environment, multiple sperm health assays were conducted. Sperm forward progressive motility assessment (Figure 2A) showed declining motility in the vaccinated group from 1 (NS compared with naïve), 2 (*P* < 0.05 vs naïve), and 3 months (*P* < 0.01 vs naïve); however, vaccination significantly improved sperm motility compared with adjuvant-only at 1 (*P* < 0.0001) and 3 months (*P* < 0.01) post-infection. At each timepoint, adjuvant-only was unable to prevent the extensive infection-induced reduced motility compared with control non-infected sperm (*P* < 0.0001).

Further markers of sperm quality were examined, first using the sperm chromatin dispersion assay, a measure of DNA integrity (Figure 2B). At all three timepoints post-infection, sperm DNA integrity was fully protected by vaccination (*P* < 0.01–0.0001) and no impact from vaccination was observed on sperm health (comparing vaccinated vs naïve = NS). Contrastingly, infection-induced damage to sperm DNA was not prevented by IMX administration alone, with significant increases above both the naïve (*P* < 0.001 – *P* < 0.0001) and the vaccinated (*P* < 0.01 – *P* < 0.0001) groups.

Next, a sperm-oocyte zona pellucida binding assay, representative of the first step of fertilization (Figure 2C), was performed. At 1 month post-infection, there was no difference between groups. At 2 months, there was a significant difference between the naïve and adjuvant-only groups (*P* < 0.05), indicating infection-induced damage, and between the adjuvant-only and vaccinated (*P* < 0.05) indicating vaccination provided protection. At 3 months, vaccination partially protected against reduced sperm-oocyte zona pellucida binding (naïve vs vaccinated = NS), but the adjuvant-only group still showed significantly reduced sperm-oocyte zona pellucida binding (naïve vs adjuvant-only = *P* < 0.05).

Finally, the sperm proteome was analyzed using SWATH-MS, which resulted in quantitation of 21 944 peptides corresponding to 29 652 proteins across different conditions (Figure 2D). When the adjuvant-only group was compared with the naïve group, 8 significantly downregulated proteins (blue dashed box) were identified including 60S ribosomal protein L27 (Rpl27; Padj = 0.0385; FC = 0.34), succinyl-CoA:3-ketoacid coenzyme A transferase 1 (Oxct1; Padj = 0.0193; FC = 0.43), calcium load-activated calcium channel (Tmco1; Padj = 0.0193; FC = 0.23), target of EGR1 protein 1 (Toe1; Padj = 0.0172; FC = 0.39), PC4 and SFRS1-interacting protein (Psip1; Padj = 0.0385; FC = 0.22), serine/arginine repetitive matrix protein 1 (Srrm1; Padj = 0.0251; FC = 0.49), solute carrier family 25 member 40 (Slc25a40; Padj = 0.0385; FC = 0.38), and small nuclear ribonucleoprotein-associated protein N (Snrpn; Padj = 0.0368; FC = 0.45). Several of these proteins are related to sperm motility, outlined in the Discussion section. When the vaccinated group was compared with the naïve group, only one protein was significantly downregulated; immediate early response 3-interacting protein 1 (Gm50364; Padj = 0.0249; FC = 0.13).

Sperm count, vitality, and morphology were also assessed (Supplementary Figure 1). No differences in sperm count were observed at any timepoint (Supplementary Figure 1A). No differences in sperm vitality were observed at 1 and 2 months post-infection, but at 3 months, adjuvant-only was insufficient to prevent viability loss (*P* < 0.05) whereas vaccinated animals showed no difference to the naïve group (Supplementary Figure 2B). Assessment of morphology showed significant vaccine mediated protection at 2 (*P* < 0.05) and 3 months (*P* < 0.0001) post-infection, the vaccinated group was no different to the non-infected controls at any timepoint, and adjuvant-only was insufficient to protect against infection-induced damage across the time course (Supplementary Figure 2C). Examples of normal and abnormal spermatozoal morphology are included (Supplementary Figure 2Cii). We also tested the level of serum testosterone to examine whether testicular health was damaged in other ways during infection, and no change was observed (Supplementary Figure 11).

### Prophylactic vaccination induces systemic T-cell cytokine expression

To begin elucidating the immunological mechanism for vaccine-mediated reduction of tissue burden and sperm damage, expression of several cytokines from splenocyte-derived T cells was examined (Supplementary Figure 9A) 1 month after infection by flow cytometry (gating displayed in Supplementary Figure 4). Results displayed are after MOMP stimulation normalized to unstimulated controls. Vaccination produced equal expression of all cytokines compared with the naïve group and increased expression of IFNγ (*P* < 0.01), TNFα (*P* < 0.0001), and IL13 (*P* < 0.001) compared with the adjuvant-only group, from CD3/CD4^+^ T cells. At this late timepoint, the adjuvant-only group had no significant expression of cytokines compared with the naïve group. For CD3/CD8^+^ T cells, vaccination induced increased expression of IFNγ (*P* < 0.01), IL13 (*P* < 0.05), IL10 (*P* < 0.01), and TGFβ (*P* < 0.01) compared with the adjuvant-only group, and increased expression of IL13 (*P* < 0.05), and IL10 (*P* < 0.01) compared with the naïve.

From CD3/CD4/Foxp3^+^ cells, presumptively regulatory T cells, vaccination-induced over-expression of IL17 (*P* < 0.05), and IL13 (*P* < 0.0001) compared with the adjuvant-only group. The adjuvant-only group had significant over-expression of IFNγ (*P* < 0.05) and under-expression of IL13 (*P* < 0.05) and TGFβ (*P* < 0.001) compared with the naïve. Vaccination also increased expression of IL13 (*P* < 0.01) and TGFβ (*P* < 0.01) over the naïve. Within CD3/CD8/Foxp3^+^ regulatory T cells, vaccination increased expression of IL13 (*P* < 0.05) over the adjuvant-only group and IFNγ (*P* < 0.05) and IL13 (*P* < 0.05) over the naïve. Both vaccinated and adjuvant-only groups had decreased IL10 (*P* < 0.0001) and TGFβ (*P* < 0.0001) expression compared both naïve.

The abundance of CD3^+^ and FOXP3^+^ cells was examined (Supplementary Figure 9B) for each sample analyzed for cytokine expression to understand whether differential representation of cell types factored into analysis. The adjuvant-only group contained significantly higher percentage of CD3^+^ (*P* < 0.01) and FOXP3^+^ (*P* < 0.05) cells and the vaccinated group contained significantly increased FOXP3^+^ (*P* < 0.05) cells. However, there was no difference in the total cells counted of either cell type.

### Prophylactic vaccination prevents pathogenic testicular CD4 T-cell influx

**Figure 3:**
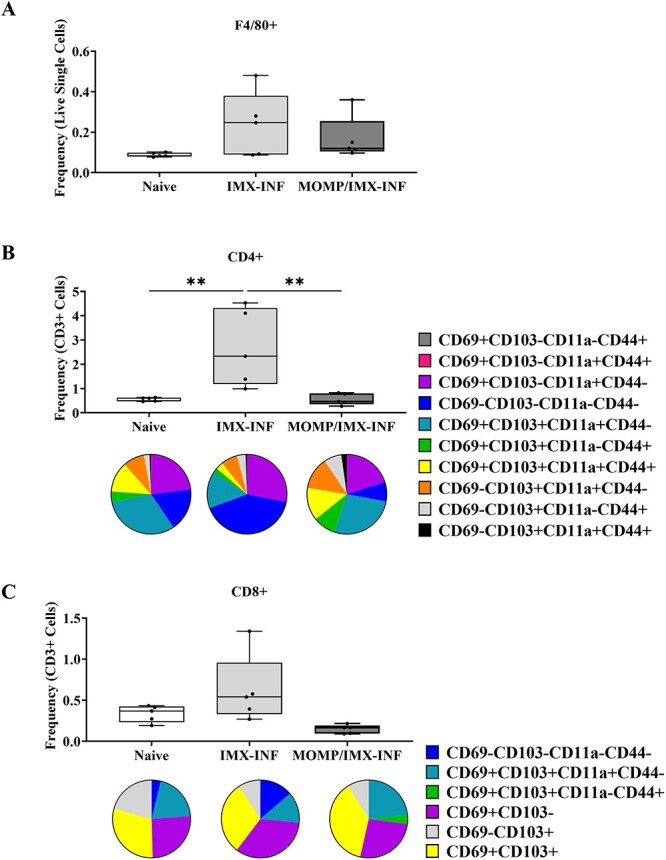
Frequency and phenotype of testicular macrophages and T cells. Mice were immunized with ISCOMATRIX (IMX) or MOMP-IMX then infected (INF) with *C. muridarum via* the intra-penile route. Three months post-infection, testes were harvested, decapsulated, and digested, and cells in the interstitial compartment were stained and analyzed by flow cytometry (Cytoflex S, Beckman Coulter). (A) Macrophages were identified by F4/80 positivity. (B) CD4 T cells were identified, and activity and residency were characterized using CD69, CD103, CD11a, and CD44. (C) CD8 T cells identified, and activity and residency were characterized using CD69, CD103, CD11a, and CD44. Graphs and statistical analyses (two-way ANOVA with Tukey’s multiple comparisons test, ^*^^*^P < 0.01) were generated in GraphPad Prism (v9.2.0).

After the examination of the systemic response, the testis specific response was investigated (Figure 3). Testes were harvest, decapsulated, and the interstitial immune cell compartment was isolated and analyzed by flow cytometry (gating shown in Supplementary Figure 5). Macrophages (Figure 3A) were quantified and there was no difference in the number between groups.

CD4 T cells (Figure 3B) were quantified next as a proportion of CD3^+^ cells, which in the testes also includes Leydig cells. There were significantly increased cells in the adjuvant-only group compared with both naïve and vaccinated (*P* < 0.01). CD4 T cells were then further characterized for tissue residency. Cells that were CD62L negative were found in all combinations of positivity and negativity of CD69, CD103, CD11a, and CD44. Phenotypic changes were present for both the adjuvant-only and vaccinated groups compared with the naïve. Predominantly, the quadruple negative phenotype (blue) was increased in the adjuvant-only group and CD69^-^ phenotypes (orange, gray, black) were increased in the vaccinated group.

Finally, CD8 T cells (Figure 3C) were quantified. There were no differences in the number of cells between groups. The CD8 T cells that were CD62L negative were also further characterized for residency phenotypes. As fewer numbers of CD8 T cells were present, only some combinations of positivity and negativity of CD69, CD103, CD11a, and CD44 were determined. Where insufficient cells were present for phenotypes to be observed in all five mice assayed, these types were not quantified. There were phenotypic changes between the three groups, notably an increased prevalence of the quadruple negative (blue) and CD69^+^CD103^-^ (purple) populations in the adjuvant-only group and switching to the presence of CD69^+^CD103^+^CD11a^-^CD44^+^ (green) and absence of quadruple negative (blue) cells in the vaccinated.

### Prophylactic vaccination induces IgG and IgA secretion

**Figure 4:**
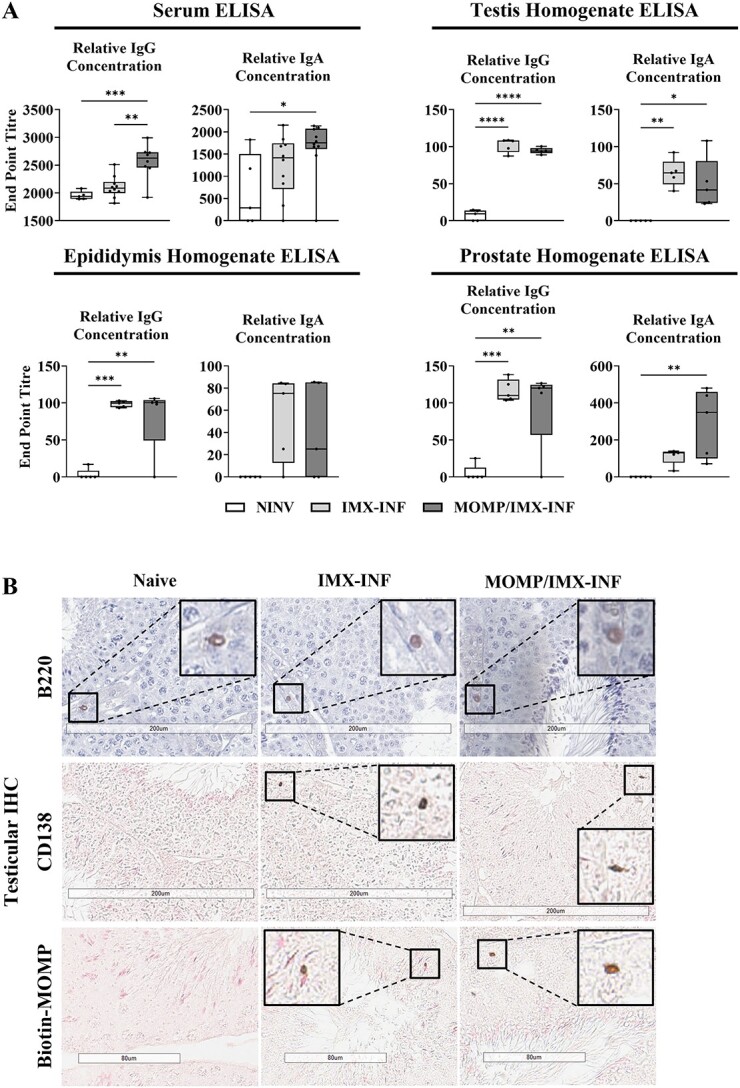
Detection of antibodies and B cells. Mice were immunized with ISCOMATRIX (IMX) or MOMP+IMX then infected (INF) with *C. muridarum via* the intra-penile route, then after 1, 2, and 3 months, sera, testes, epididymides, and prostates were harvested (*n* = 5/group). (A) ELISA was performed on sera and tissue homogenates for the 3-month timepoint for MOMP specific IgG and IgA end point titers. (B) Immunohistochemistry was performed to confirm the presence of tissue (testis) specific B cells. Immature B cell marker B220, mature B cell marker CD138, and MOMP-specific antibodies were detected. Staining was developed using DAB, visualized as dark brown coloration, and slides were counterstained using hematoxylin (purple) or nuclear fast read (pink). Slides were scanned (Aperio AT Turbo, Leica) and visualized (Aperio ImageScope v12.4.3.5008), images representative of *n* = 5 mice, scale bar = 200 or 80 μm.

B cells and antibody secretion were also investigated as part of the vaccine mechanism of action. After vaccination and infection, serum and homogenates of testis, epididymis, and prostate were assayed for the presence of IgG and IgA *via* MOMP-specific ELISA (Figure 4A). Serum contained significantly elevated titers of IgG in the vaccine group compared with the naïve (*P* < 0.001) and adjuvant-only groups (*P* < 0.01), and also significantly elevated titers of IgA in the vaccine group compared with the naïve (*P* < 0.05) but not adjuvant-only group.

Within testis homogenates, adjuvant-alone generated MOMP-specific IgG (*P* < 0.0001) and IgA (*P* < 0.01) and vaccination generated both IgG (*P* < 0.0001) and IgA (*P* < 0.05). Within epididymis homogenates, adjuvant-alone application generated both IgG (*P* < 0.001) and some IgA (NS) and vaccination generated some IgG (*P* < 0.01) and some IgA (NS). Prostate homogenates contained some IgG for both adjuvant-alone (*P* < 0.001) and vaccination (*P* < 0.01) and IgA significantly for the vaccine (*P* < 0.01) but not for the adjuvant-only (NS).

Next, as the important site of spermatogenesis the testicular tissue was examined further. Testicular tissue sections were investigated using IHC (Figure 4B, representative images) for B220 (immature B cells), which were found in all groups localized predominantly to testicular capillaries or in close apposition to capillaries in the interstitial space. B220 staining was validated on unrelated testis and gut tissues (Supplementary Figure 3). Next, CD138 (mature antibody secreting cells, ASC) were investigated and found rarely in the naïve group, but more frequently in the vaccinated and adjuvant-only groups, most frequently within seminiferous tubule epithelium. Among these ASCs were MOMP-specific cells, found only in the vaccinated and adjuvant-only groups in equal numbers, and not in the naïve. Testicular interstitial cells were also assayed by flow cytometry to identify CD19^+^ B cells, and results mirrored the IHC in that no significant difference in the number of positive cells was found between groups (data not shown).

### Therapeutic vaccination reduces reproductive tissue *Chlamydia* burden

**Figure 5:**
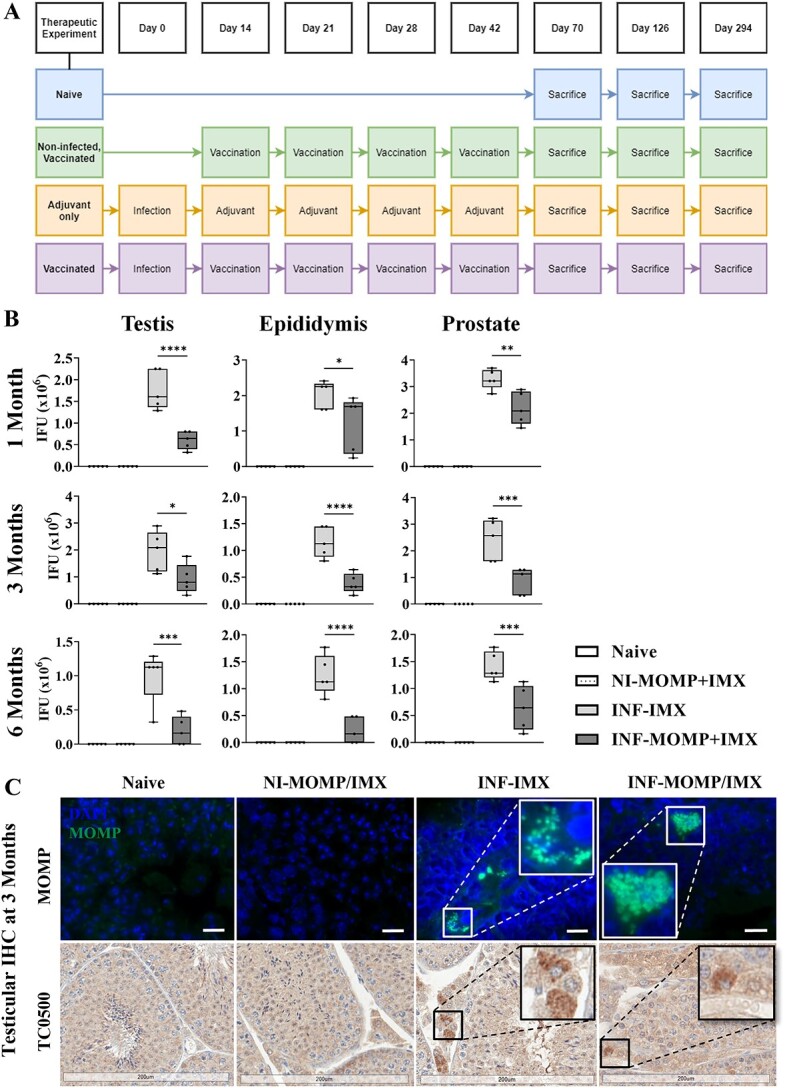
Chlamydial burden in male reproductive tissues. Mice were infected (INF) with *C. muridarum via* the intra-penile route (or mock-infected with SPG, NI) then immunized with ISCOMATRIX (IMX) or MOMP+IMX per the timeline in (A). *Chlamydia muridarum* was found in (B) the testes, epididymis, and prostates by culture of live infection from tissue homogenates and (C) the testes by immunohistochemistry (IHC) to confirm the culture results (images shown at 3 months). The IHC included MOMP (green) counterstained with DAPI (blue) where the scale bar = 10 μm and representative images were captured on X100 magnification (Axio Vert.A1, Zeiss), and active replication marker TC0500 (DAB, brown) counterstained with hematoxylin (purple) where the scale bar = 200 μm and slides were scanned (Aperio AT Turbo, Leica) and visualized (Aperio ImageScope v12.4.3.5008). Graphs and statistical analyses (two-way ANOVA with Tukey’s multiple comparisons test, ^*^P < 0.05, ^*^^*^P < 0.01, ^*^^*^^*^^*^P < 0.0001) were generated in GraphPad Prism (v9.2.0), data are mean ± SD.

We next examined whether the protection elicited by prophylactic vaccination could be replicated in a therapeutic vaccine model, where vaccination is administered after infection rather than before infection (Figure 5A). First, tissue burdens were investigated within the male reproductive tract by culture of tissue homogenates on McCoy cells.

The number of IFU per testis at 1 (adjuvant-only average = 1.786 × 10^6^ IFU; vaccinated average = 0.6109 × 10^6^; *P* < 0.0001, 65% decrease), 3 (adjuvant-only average = 1.961 × 10^6^ IFU; vaccinated average = 0.9298 × 10^6^; *P* < 0.05, 52% decrease), and 6 months (adjuvant-only average = 0.9967 × 10^6^ IFU; vaccinated = 0.1929 × 10^6^; *P* < 0.001, 80% decrease) was significantly reduced including two testes that were clear of infection at 6 months (Figure 5B).

The number of IFU per epididymis at 1 (adjuvant-only average = 2.025 × 10^6^ IFU; vaccinated average = 1.206 × 10^6^; *P* < 0.05, 40% decrease), 3 (adjuvant-only average = 1.157 × 10^6^ IFU; vaccinated average = 0.3858 × 10^6^; *P* < 0.0001, 66% decrease), and 6 months (adjuvant-only average = 1.254 × 10^6^ IFU; vaccinated average = 0.2251 × 10^6^; *P* < 0.0001, 82% decrease) was significantly reduced including two epididymides that were clear of infection at 6 months (Figure 5B).

The number of IFU per prostate at 1 (adjuvant-only average = 3.279 × 10^6^ IFU; vaccinated average = 2.186 × 10^6^; *P* < 0.01, 33% decrease), 3 (adjuvant-only average = 2.411 × 10^6^ IFU; vaccinated average = 0.8681 × 10^6^; *P* < 0.001, 63% decrease), and 6 months (adjuvant-only average = 1.415 × 10^6^ IFU; vaccinated average = 0.6430 × 10^6^; *P* < 0.01 to −0.001, 54% decrease) was also significantly reduced.

As expected, no IFU or inclusions were found in the tissues of the naïve and non-infected- vaccinated groups. The presence of *Chlamydia* within the testes was again confirmed with IHC (Figure 5C) for MOMP and TC0500, indicating actively replicating inclusions were present.

### Therapeutic vaccination protects or partially protects sperm health

**Figure 6:**
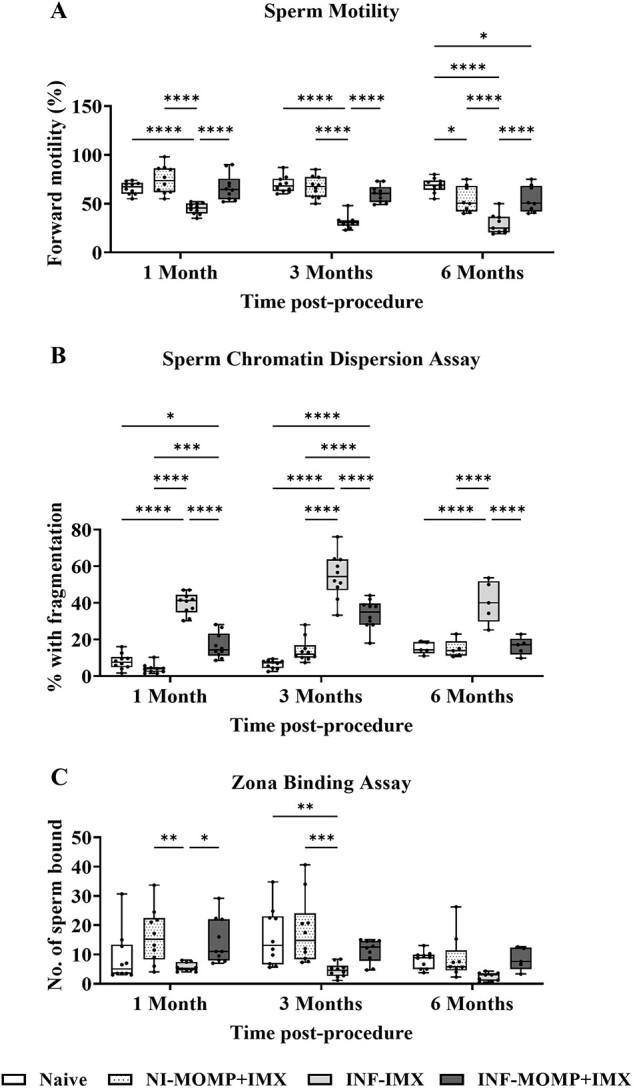
Sperm motility, chromatin dispersion, and oocyte-binding during *C. muridarum* infection and therapeutic vaccination. Intra-penile *C. muridarum* infection (INF) or mock with SPG (NI) was followed by immunization with ISCOMATRIX (IMX) alone or MOMP mixed with IMX, then after 1, 3, and 6 months, sperm were harvested from the vas deferens of all mice (*n* = 10/group). Sperm were imaged using brightfield microscopy (Axio Ver.A1, Zeiss). (A) There were differences in sperm motility between groups at all timepoints. (B) There were differences in sperm chromatin dispersion between groups at all timepoints. (C) There were differences in oocyte-binding between groups at 1 and 3 months. Graphs and statistical analyses (two-way ANOVA with Tukey’s multiple comparisons test, ^*^P < 0.05, ^*^^*^P < 0.01, ^*^^*^^*^P < 0.001, ^*^^*^^*^^*^P < 0.0001) were generated in GraphPad Prism (v9.2.0), data are mean ± SD.

As therapeutic vaccination reduced chlamydial burden we also determined if vaccination protected sperm health. Vaccination protected sperm motility (Figure 6A) at 1 and 2 months compared with adjuvant-only (*P* < 0.0001) and partially protected motility at 3 months with significant improvement compared with adjuvant-only (*P* < 0.0001), but small reduction compared with the naïve group (*P* < 0.05). Vaccination partially prevented sperm DNA damage (Figure 6B) at 1 and 3 months with significant improvement compared with the adjuvant-only (*P* < 0.0001) but with more than the naïve group (1 month: *P* < 0.05; 3 months: *P* < 0.0001). However, at 6 months, vaccination fully prevented DNA damage with no difference between the vaccinated and naïve/non-infected-vaccinated groups and infection treated with adjuvant-only maintaining significant damage compared with all other groups (*P* < 0.0001). Next, vaccination partially protected sperm-oocyte zona pellucida binding (Figure 6C) at 1 and 3 months, but at 6 months , no difference was observed between any groups, although the adjuvant-only group was lowest, likely due to age-related sperm degeneration.

Sperm count, vitality, and morphology were again also assessed (Supplementary Figure 2). No differences in sperm count were observed at any timepoint (Supplementary Figure 2A). No differences in sperm vitality were observed at 1 month. At both 2 (*P* < 0.01) and 3 months (*P* < 0.05) post-treatment, the adjuvant-only was insufficient to prevent viability loss, whereas in vaccinated animals, viability was no different to the naïve and non-infected-vaccinated groups (Supplementary Figure 2B). Assessment of morphology showed no difference between groups at 1 month (Supplementary Figure 2C). Significant abnormal morphology was observed in the adjuvant-only group at 3 months (*P* < 0.0001) but not in vaccinated animals, indicating vaccine mediated protection. At 6 months post-treatment, abnormal morphology increased in all groups, although it was lowest in the vaccinated animals (vaccinated group compared with non-infected-vaccinated; *P* < 0.01), potentially indicating age related degeneration.

### Therapeutic vaccination induces systemic T-cell cytokine expression

To understand the immunological mechanism for vaccine-mediated protection, T cells were again examined after in vitro stimulation with MOMP (Supplementary Figure 10A). Differences in IL10 expression (vaccination increased compared with adjuvant-only and non-infected-vaccinated, *P* < 0.05) and TGFβ (adjuvant-only decreased compared with naïve, *P* < 0.05) were observed for CD4^+^ T cells. Differences in TNFα (vaccination increased compared with naïve and non-infected-vaccinated, *P* < 0.05), IL10 (vaccinated compared with all groups, *P* < 0.05), and TGFβ (adjuvant-only decreased compared with non-infected-vaccinated, *P* < 0.05) were observed for CD8^+^ T cells. Differences in IL10 (vaccination increased compared with non-infected-vaccinated, *P* < 0.05) were observed for CD4^+^ Treg cells. There was no change to cytokine expression between any groups for CD8^+^ Treg cells.

The abundance of CD3^+^ and FOXP3^+^ cells was examined (Supplementary Figure 10B) for each sample analyzed for cytokine expression to understand whether differential representation of cell types factored into analysis. The adjuvant-only group contained significantly lower percentage of CD3^+^ (*P* < 0.01) cells and there was no difference in the percentage or total cells counted of either cell type for any other groups.

### Therapeutic vaccination induces testicular immunological homeostasis

**Figure 7:**
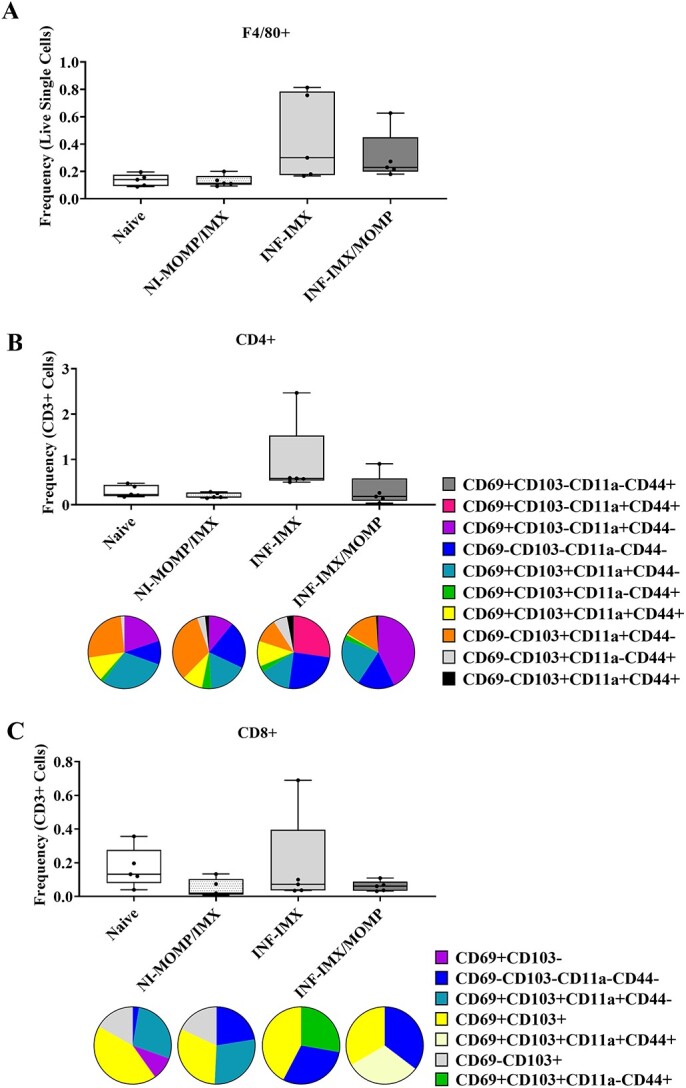
Frequency and phenotype of testicular macrophages and T cells. Intra-penile *C. muridarum* infection (INF) or mock with SPG (NI) was followed by immunization with ISCOMATRIX (IMX) alone or MOMP mixed with IMX. Three months post-infection, testes were harvested, decapsulated and digested, and cells in the interstitial compartment were stained and analyzed by flow cytometry (Cytoflex S, Beckman Coulter). (A) Macrophages were identified by F4/80 positivity. (B) CD4 T cells were identified, and activity and residency were characterized using CD69, CD103, CD11a, and CD44. (C) CD8 T cells identified, and activity and residency were characterized using CD69, CD103, CD11a, and CD44. Graphs and statistical analyses (two-way ANOVA with Tukey’s multiple comparisons test, ^*^^*^P < 0.01) were generated in GraphPad Prism (v9.2.0).

After the examination of the systemic response, the testis specific response was investigated (Figure 7). Testes were harvest, decapsulated, and the interstitial cell compartment was isolated. Macrophages (Figure 7A) were quantified and there was no difference in the total number between groups.

CD4^+^ T cells (Figure 7B) were quantified next as a proportion of CD3^+^ cells, which, in the testis, also includes Leydig cells. There was no difference in the total number of cells between groups. CD4 T cells were then further phenotyped for tissue residency. When delineating combinations of positivity and negativity of CD69, CD103, CD11a, and CD44, phenotypic differences were clear between all groups. Vaccination alone caused small changes in abundance of all types. Treatment with adjuvant-only caused major changes including influx of CD69^+^CD103^−^CD11a^+^CD44^+^ (pink) cells. Therapeutic vaccination also caused widespread changes, including a marked increase in CD69^+^CD103^−^CD11a^+^CD44^−^ (purple) cell abundance.

Finally, CD8^+^ T cells (Figure 7C) were quantified. There were no differences in the total number of cells between groups. CD8 T cells were then further phenotyped for tissue residency. As fewer numbers of CD8^+^ T cells were present, only some combinations of positivity and negativity of CD69, CD103, CD11a, and CD44 were determined. Where insufficient cells were present for phenotypes to be observed in all five mice assayed, these types were not quantified. There were phenotypic changes between all groups, notably, the absence of the CD69^+^CD103^−^ (purple) and increased abundance in quadruple negative (blue) types in all compared with the naïve. The adjuvant-only group contained a CD69^+^CD103^+^CD11a^−^CD44^+^ (green) population and the vaccinated group instead contained CD69^+^CD103^+^CD11a^+^CD44^+^ (light yellow) quadruple positive population.

### Therapeutic vaccination induces IgG and IgA secretion

**Figure 8:**
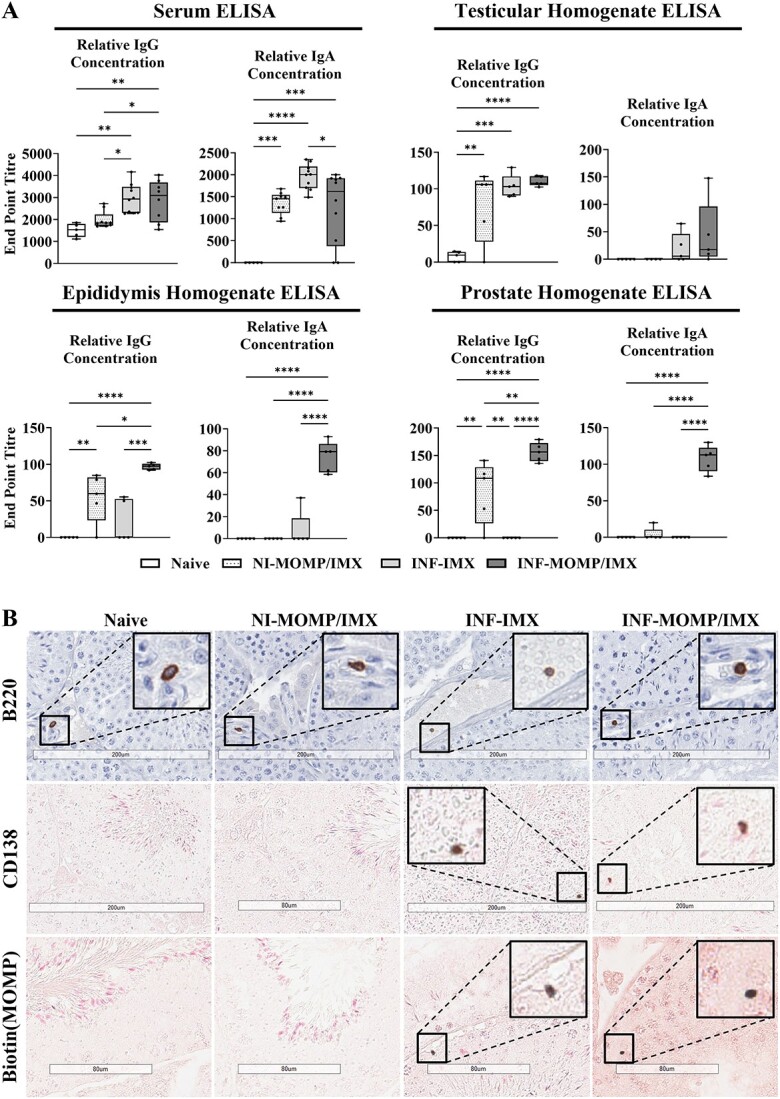
Detection of antibodies and B cells. Intra-penile *C. muridarum* infection (INF) or mock with SPG (NI) was followed by immunization with ISCOMATRIX (IMX) alone or MOMP mixed with IMX, then after 3 months, sera, testes, epididymides, and prostates were harvested (*n* = 5/group). (A) ELISA was performed on sera and tissue homogenates for the 3-month timepoint for MOMP specific IgG and IgA end point titers. (B) Immunohistochemistry was performed to confirm the presence of tissue (testis) specific B cells at 3 months. Immature B cell marker B220, mature B cell marker CD138, and MOMP specific antibodies were detected. Staining was developed using DAB, visualized as dark brown coloration, and slides were counterstained using hematoxylin (purple) or nuclear fast read (pink). Slides were scanned (Aperio AT Turbo, Leica) and visualized (Aperio ImageScope v12.4.3.5008), images representative of *n* = 5 mice, scale bar = 200 or 80 μm. Graphs and statistical analyses (two-way ANOVA with Tukey’s multiple comparisons test, ^*^^*^P < 0.01) were generated in GraphPad Prism (v9.2.0).

B cells and antibody secretions were again investigated as part of the therapeutic vaccine mechanism of action. First, serum and homogenates of testis, epididymis, and prostate were assayed for the presence of IgG and IgA *via* MOMP-specific ELISA (Figure 8A). In serum, the non-infected-vaccinated group had minimal IgG (NS) but significant IgA (*P* < 0.001). Both the infected groups generated significantly more IgG (*P* < 0.05) than the non-infected, but similar levels of IgA (NS). Both infected groups also generated significant IgG (*P* < 0.01) and IgA (adjuvant-only = *P* < 0.0001; vaccinated = *P* < 0.001) compared with the naïve, although the vaccine group elicited less IgA than the adjuvant-only (*P* < 0.05).

In testicular homogenates, MOMP-specific IgG was found in significant titers in all of the non-infected-vaccinated (*P* < 0.01), adjuvant-only (*P* < 0.001), and vaccinated (*P* < 0.0001) groups compared with the naïve. Some IgA was also found within the two infected group homogenates but not at significant titers. For epididymis homogenates, vaccination produced the highest titers of IgG (vs naïve = *P* < 0.0001; non-infected-vaccinated = *P* < 0.05; adjuvant-only = *P* < 0.001) and IgA (vs naïve = *P* < 0.0001; non-infected-vaccinated = *P* < 0.0001; adjuvant-only = *P* < 0.0001). However, IgG but not IgA was still detected compared with the naïve for the non-infected-vaccinated (*P* < 0.01) and adjuvant-only (NS) groups. For prostate homogenates, the same trend as the epididymis was present. No IgG or IgA was elicited in the adjuvant-only group. IgG was detected in the non-infected-vaccinated group compared with all other groups (*P* < 0.01), but no IgA. However, the vaccinated group had the highest IgG (*P* < 0.0001) and IgA (*P* < 0.0001) compared with both the naïve and adjuvant-only groups.

Next, the testicular tissue sections were investigated using IHC (Figure 8B, representative images) for B220 (immature B cells), which were found in all groups localized predominantly to testicular blood vessels or in close apposition to vessels in the interstitial space. Next, ASCs were investigated using CD138 and biotin-MOMP and found frequently in both the vaccinated and adjuvant-only groups predominantly in the seminiferous tubule epithelium. CD138-positive cells were found rarely in the naïve and non-infected-vaccinated groups and no MOMP-specific ASCs were found in those groups.

## Discussion

Our findings elucidate, for the first time, the long-term response to male mouse chlamydial vaccination, which significantly reduced and, in some cases, cleared chlamydial burden from the prostates, epididymides, and testes. Vaccination moderated testicular immunological responses to both attenuate pathology while protecting important aspects of sperm function. The testes, as the site of spermatogenesis, are under strict immunological control to limit exposure to auto-immunogenic sperm, which begins production in adolescence well after tolerance to self is established, creating a risk to spermatogenesis when eliciting a strong testicular specific response to infection.

Pro-inflammatory immune cell infiltration has a detrimental impact on testicular structure and production of quality sperm [45]. The well-established model of experimental autoimmune orchitis (EAO) exemplifies this, with extensive infiltration of autoreactive CD4 T cells within the interstitial compartment causing extensive damage to seminiferous tubules and depletion of spermatogenic cells [46]. The adjuvant-only group in the prophylactic model closely mirrors that scenario, whereas the trend was less pronounced in the therapeutic model owing to prior insult from infection in both the adjuvant-only and vaccine groups. Our vaccine prevented T-cell influx, and consequent pathology associated with orchitis, likely resulting in the maintenance of tolerance to sperm. The deleterious impact of *Chlamydia* and the protective effect of vaccination on the endogenous sperm proteome was clear in the prophylactic model. Dysregulation of proteins including Rpl27, Oxct1, and Snrpn, as seen here, associates with human sperm immobility and infertility, which not only provides a molecular quantifier for vaccine-mediated protection of sperm but also shows the mouse model mimics the human disease state, useful in studying the Chlamydial disease and vaccine development.

Low levels of Chlamydia remained in the testes of vaccinated mice demonstrating that sterile immunity was not achieved, a possible trade-off for maintenance of immune privilege. This is consistent with the ongoing issue in the chlamydial field where a dichotomy of choosing to protect against pathology is paramount, requiring a different immune response than that needed to clear infection [47]. Like the EAO model, Tregs were identified within the interstitial compartment, which may contribute to this tolerance of infection and infection-induced damage; however, no differences in number of IL10 secreting Tregs were observed between groups. The IL10 being produced, significantly in the therapeutically vaccinated mice (Supplementary Figure 6), instead comes from an alternate source, likely resident testicular macrophages that normally have an anti-inflammatory phenotype to protect tissue homeostasis and limit damage [48–50]. We surmise that therapeutic vaccination-induced immunity controls further chlamydial dissemination to the testes from the penile urethra, in which infection persisted for more than 3 months (Supplementary Figure 7), which allows resident testicular macrophages the opportunity to enable the repair of infection-induced pathology, likely through IL10 production that is essential for epithelial protection and repair as seen in other tissues [51]. We examined the presence of testicular macrophages in the interstitial compartment. As the total number of cells did not increase, this suggests possible phenotypic changes to compensate for inflammatory processes occurring within the testes.

Similarly, testicular T cells underwent phenotype flux, the most telling of which was expansion of the quadruple negative CD4^+^ T cells in the adjuvant-only group. Although tissue resident T cells have highly variable expression of CD69, CD103, CD11a, and CD44 based on location and function, including quadruple negative cells in intestinal lamina propria for example [52], we hypothesize that the observed population represents infiltrating cells rather than proliferation of a resident phenotype, as per the EAO model. This bias toward T-cell infiltration is supported by the CD8^+^ data, in which a phenotype (CD69^+^CD103^+^CD11a^−^CD44^+^) appears only in the vaccinated mice, likely indicating entry of this cell type earlier in the immune response. Development of phenotypically diverse resident T cells in response to infection is documented [52], including in the testis, although the diversity of types seen here has not been reported previously [53].

The therapeutic model demonstrated similarly broad changes in the immune cell compartment. There were no changes in the number of macrophages or T cells between any groups. Completely opposite to the prophylactic model, protection associated with CD69^+^ macrophages, with more than 75% of cells having this phenotype. Two types of testicular macrophages exist with distinct origins, the first a self-renewing type seeded during embryogenesis, and the second recruited and replenished from the circulatory system [49]. Both are polarized to an anti-inflammatory phenotype, critical for testicular function. So, these results may represent the activation of existing bone-marrow derived macrophages, to combat infection having now been primed and directed by the vaccination. We hypothesize that macrophages mediate the long-term clearance of infection in both vaccination models. Typically, testis with healthy functioning macrophages have limited T-cell activation capability and truncated pro-inflammatory cytokine expression [49] while still retaining some bactericidal activity [54].

The considerable diversity of testicular T-cell phenotypes was observed again in the therapeutic experiment. Expansion of the CD4^+^CD69^+^CD103^-^CD11a^+^CD44^−^ and CD8^+^CD69^+^CD103^+^CD11a^+^CD44^+^ phenotypes may be protective, as these were induced by vaccination. Additionally, the CD4^+^CD103^−^CD11a^+^CD44^+^ and CD8^+^CD69^+^CD103^+^CD11a^−^CD44^+^ phenotypes may be pathogenic, as these cells were increased in the adjuvant-only group. It seems likely these types of cells respond to the testicular infection and were re-directed appropriately by vaccination but inappropriately by the non-specific adjuvant application, and much like the prophylactic model, therapeutic administration of the adjuvant is insufficient to protect the testicular environment.

We also assessed the systemic response by splenic T cells expressing cytokines with known importance to resolution of chlamydial infections and pathology; IFNγ, TNFα, IL17, IL13, IL10. *Chlamydia* infection consistently coincided with the lack of IL10 and TGFβ expressing cells, conversely to the vaccination groups, indicating vaccination upregulates an anti-inflammatory/tissue repair type response. This may also act as a counterbalance to pro-inflammatory IFNγ and TNFα that were also upregulated by vaccination, both of which play a role in the clearance of infection but also in pathology development [55, 56]. Induction of this balanced immune response likely attenuates pathology usually associated with infection, including scar tissue formation, thereby maintaining spermatogenesis.

IL13 was also affected by vaccination. The role of IL13 in chlamydial infections is unclear, particularly for males. It has been shown that macrophages from IL13−/− mice phagocytosed greater numbers of UV-killed *C. muridarum* than WT macrophages [57], which, in the context of our study, could mean vaccine-mediated IL13 secretion make macrophages refractory to *C. muridarum* uptake in the urethra/testis, depleting the infection reservoir and decreasing disease severity. In female mice, co-expression of IFNγ and IL13 from a subpopulation of CD4 T cells was also protective against ovarian pathology [58], and there may be a similar mechanism for males. CD8 T cells have conflicting roles in chlamydial infections, having been shown to cause immunopathology, predominantly through the production of TNFα [59], but in some cases also protection through atypical *Chlamydia*-specific cells secreting IL13 in addition to TNFα, IFNγ, and IL10 [60, 61]. Collectively, literature suggests that early innate cell production of IL13 may enhance chlamydial infection-induced pathology but later in infection, when the adaptive immune response is activated IL13 may be produced by both CD4 and CD8 cells, as seen in our study, to prevent or limit inflammatory pathology.

IL17 has similarly conflicting literature, with studies showing IL17 to be pathogenic and protective [62, 63], likely dependent on the context and cellular source of IL17 production. Th17 cell plasticity may allow development into protective Th1 cells during culture [64], and with IL23 in the local cytokine environment in tissue [65, 66]. Although these responses will be beneficial for control of *Chlamydia* systemically and within the lower tract, the testis would require an alternate approach, potentially in line with TGFβ and IL21-mediated reprogramming of classical Th17 cells into anti-inflammatory regulatory Th17 cells [65, 66]. Each potential differentiation pathway can certainly influence the outcome of chlamydial infection, and the duality of Th17/IL17 being both protective and pro-inflammatory may be related to, or derived from, the diverse patterns of cytokine secretion observed in vaccine-induced systemic T-cell responses previously and in this study. The T-cell response may only provide limited protection, with the suppressive environment in the testes likely favoring the anti-inflammatory pathway with constitutive expression of IL10 and TGFβ, but perhaps this limits the infection sufficiently to allow testicular macrophages time to act.

We also examined the role of B cells and antibodies in the anti-chlamydial response. Although we examined the 3-month post-procedure timepoint for both the prophylactic and therapeutic models, different responses were observed. For the prophylactic experiment, the titers of IgA and IgG were relatively equal in all bar serum IgG, where vaccination generated a significantly higher concentration. In the therapeutic model, antibody responses were remarkably different. The adjuvant-only group failed to generate IgG and IgA in many cases, which may indicate the propensity of natural infection to induce no to low-titer antibodies in the male tract. It seems prophylactic priming with either adjuvant-alone or vaccination is sufficient to overcome this, although adjuvant-alone is insufficient for pathology prevention. When we assay the 1-month post-vaccination titers in the prostate (Supplementary Figure 8), we gain a deeper understanding, that natural infection generates a short-lived low titer response, whereas vaccination provides the long-lived IgG response required for ongoing control of infection and protection.

The protection afforded by these antibodies is likely multifaceted. We see high titers in the prostates in both vaccine models, representative of the lower urogenital tract. We posit that these antibodies contribute heavily to clearance of infection, as previously characterized in poly-Ig-receptor knockout mice which had rampant infection correlating with no transport of IgA into the male tract [67]. Supplementary Figure 6 supports this idea, showing dramatically reduced numbers of chlamydial inclusions in the penile urethral epithelium (arrows) in the vaccinated groups. Alongside plentiful inclusions within the penile tissues, the adjuvant-only groups also had greatly thickened urethral epithelium indicative of ongoing inflammation. We have previously demonstrated that macrophages take up penile infection and rapidly disseminate to the testis *via* the circulatory system [6]. Vaccination may abrogate this dissemination, as well as any slower cell-cell ascending route of infection, so the upper tract is able to control and eventually eliminate the low-grade infection following initial spread to the testes. Clearance of infection is aided or likely affected by tissue resident macrophages and T cells in the testes.

In summary, we investigated the long-term repercussions of male chlamydial infection and vaccination and prevention of infection-induced pathology by vaccination, which represents significant progress in the chlamydial research field and brings male chlamydial research into closer alignment with female chlamydial research. We currently hypothesize that high antibody titers in the serum and lower reproductive tract contribute to control of infectious burden [67] and reducing/eliminating the penile chlamydial reservoir prevents continuous dissemination to the testes. The resident testicular immune cells, which are programmed to have a moderate response to infection, work to clear the testicular burden over time while still protecting spermatogenesis and fertility. Finally, vaccination supports healthy testicular function with protective resident Trem phenotypes and preventing pathogenic CD4 T-cell influx.

## Supplementary Material

Supplementary_Figure_1_ioad021

Supplementary_Figure_2_ioad021

Supplementary_Figure_3_ioad021

Supplementary_Figure_4_ioad021

Supplementary_Figure_5_ioad021

Supplementary_Figure_6_ioad021

Supplementary_Figure_7_ioad021

Supplementary_Figure_8_ioad021

Supplementary_Figure_9_ioad021

Supplementary_Figure_10_ioad021

Supplementary_Figure_11_ioad021

Supplementary_Table_1_ioad021

Supplementary_Table_2_ioad021

## Data Availability

The data underlying this article are available in the article and in its online supplementary material.
