# Supplementary figures and images for: Prophylactic and therapeutic vaccination protects sperm health from Chlamydia muridarum-induced abnormalities
Source: Biol Reprod. 2023 Feb 17;108(5):758–77. doi: 10.1093/biolre/ioad021 (PMC10183362; doi:10.1093/biolre/ioad021)

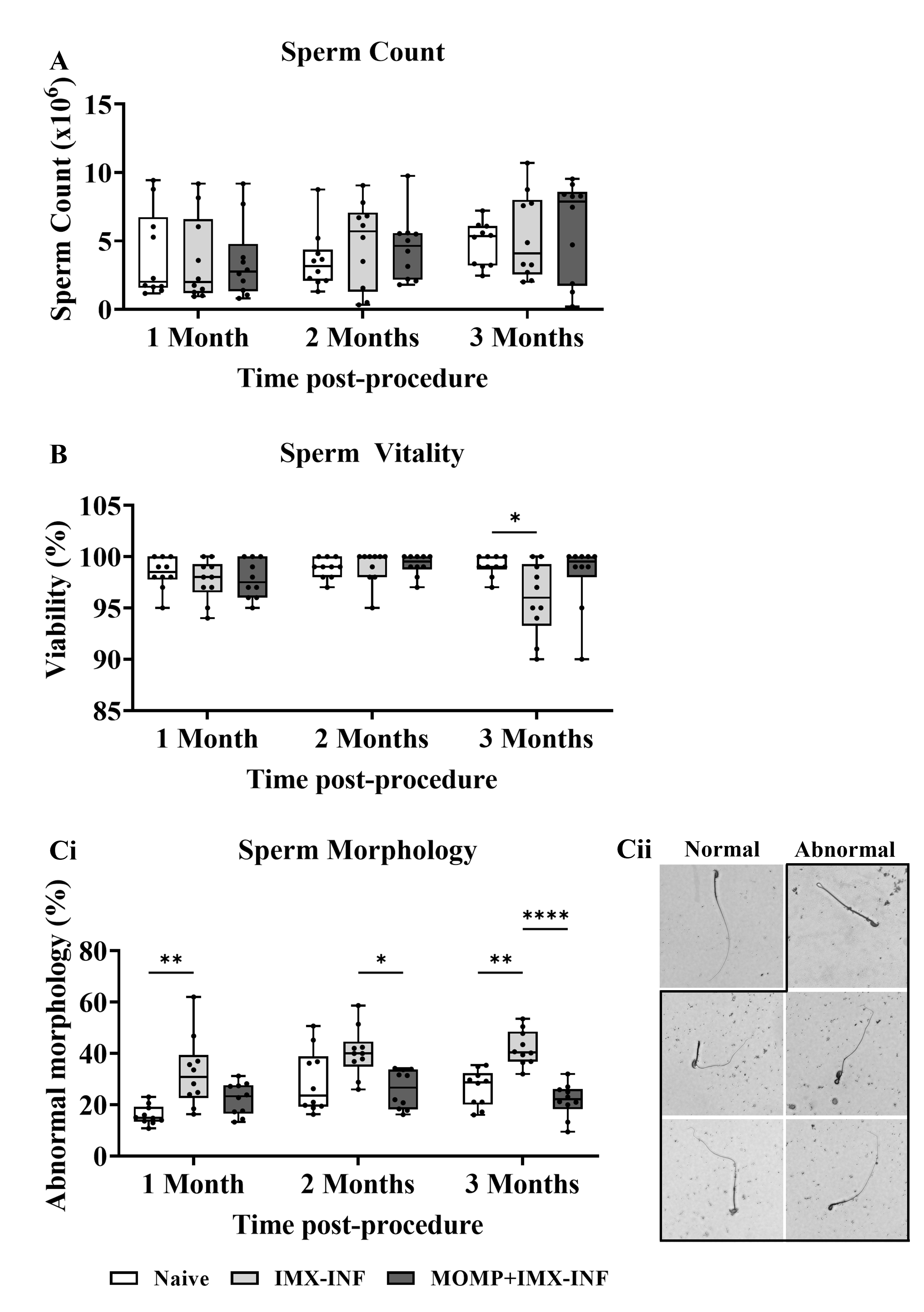

Supplement: Supplementary_Figure_1_ioad021 [file supplementary_figure_1_ioad021.zip › Supplementary_Figure_1_ioad021.tif]

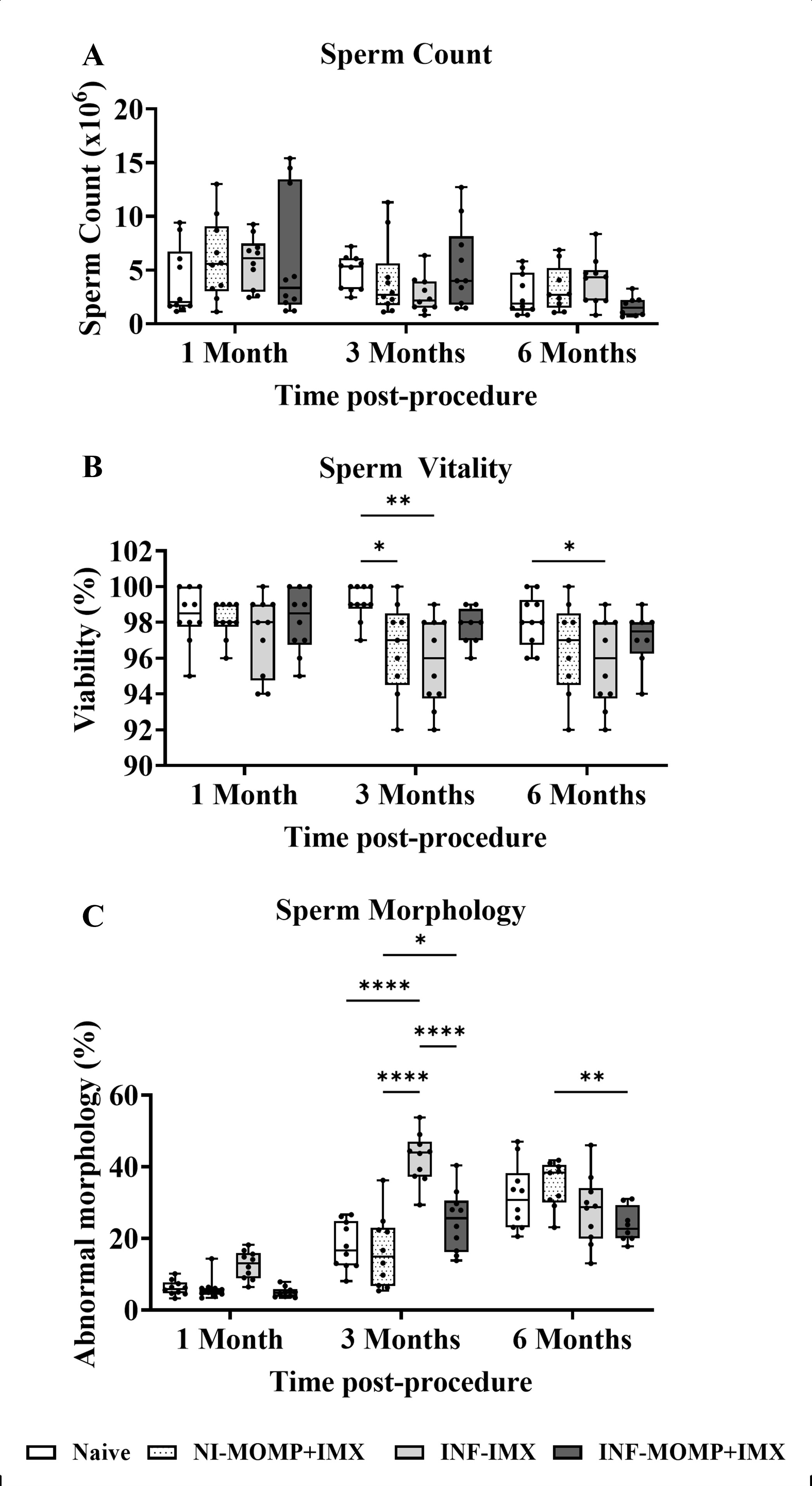

Supplement: Supplementary_Figure_2_ioad021 [file supplementary_figure_2_ioad021.jpeg]

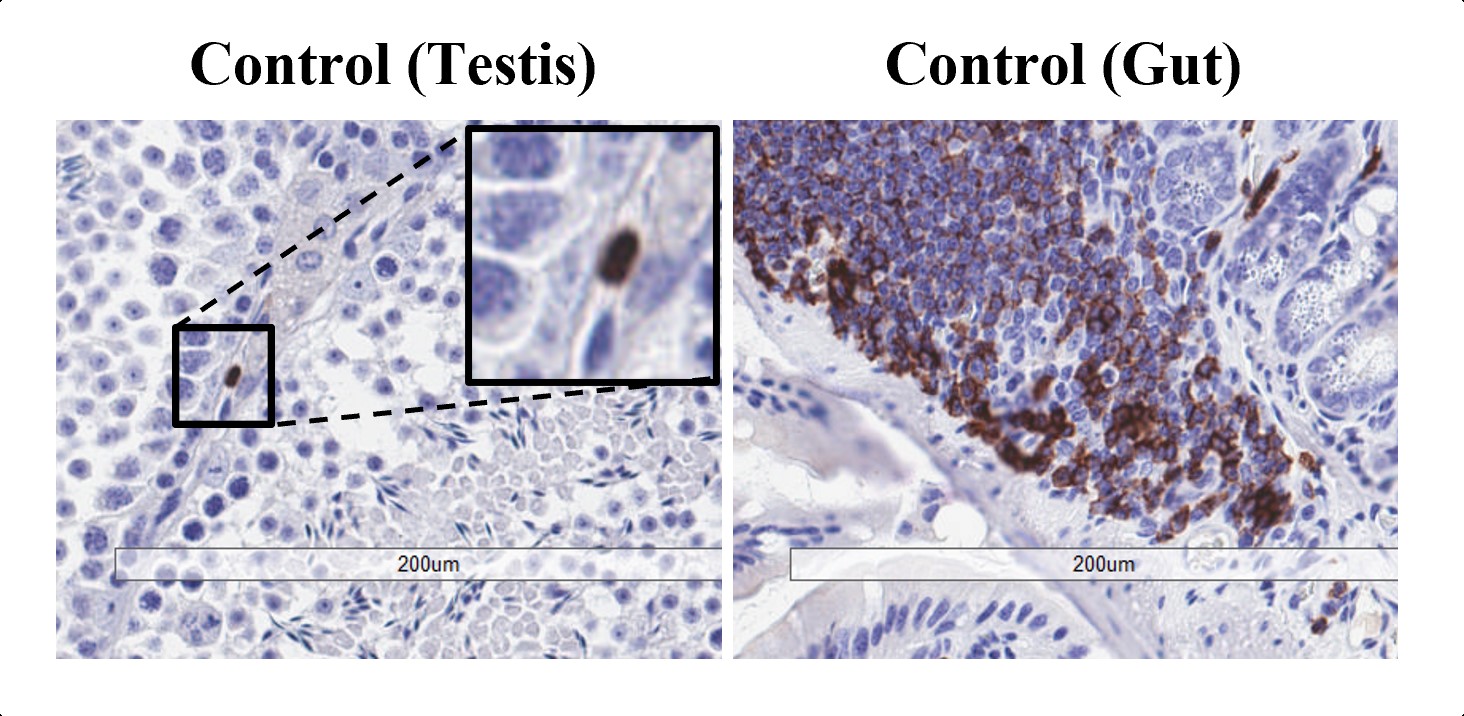

Supplement: Supplementary_Figure_3_ioad021 [file supplementary_figure_3_ioad021.jpeg]

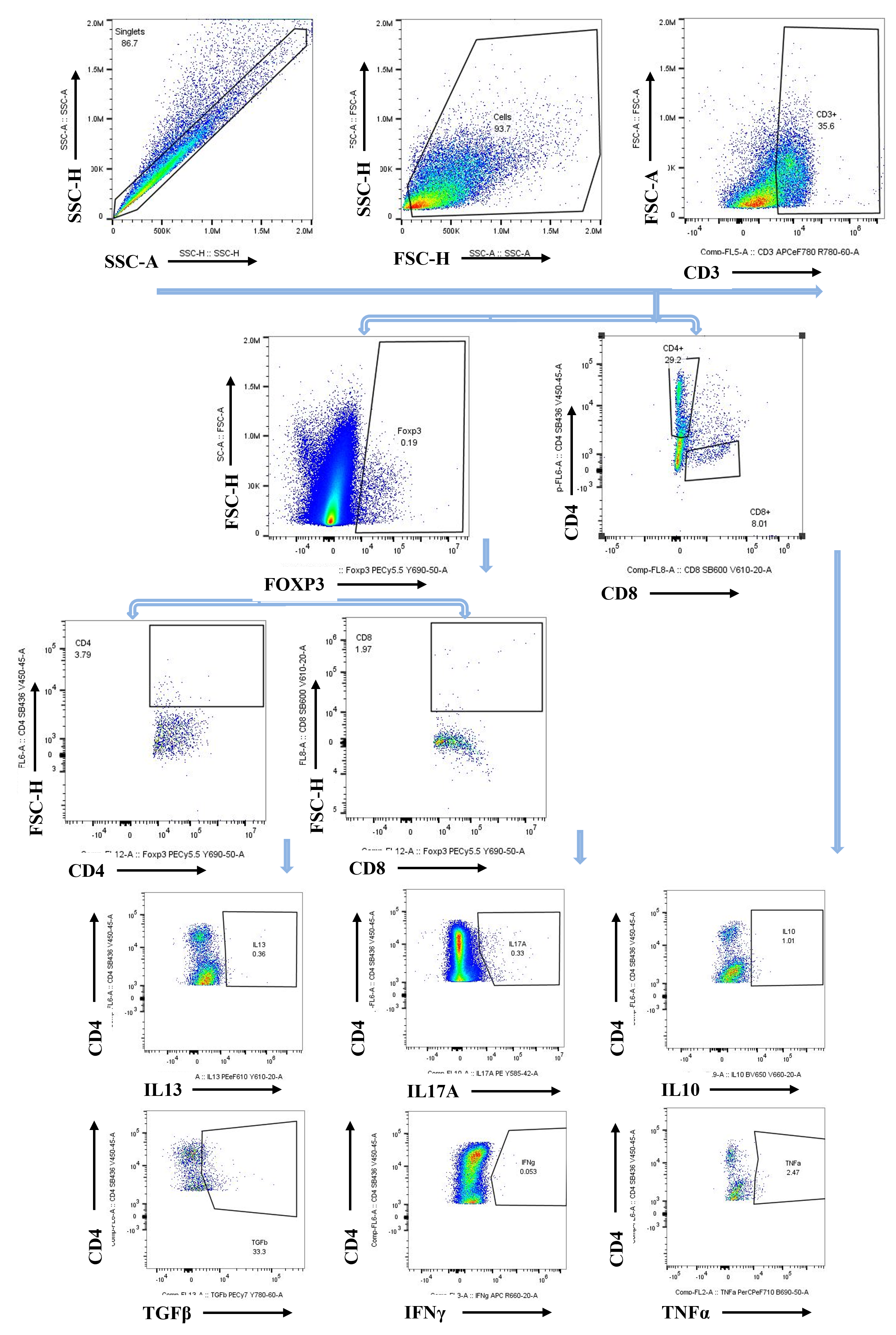

Supplement: Supplementary_Figure_4_ioad021 [file supplementary_figure_4_ioad021.zip › Supplementary_Figure_4_ioad021.tif]

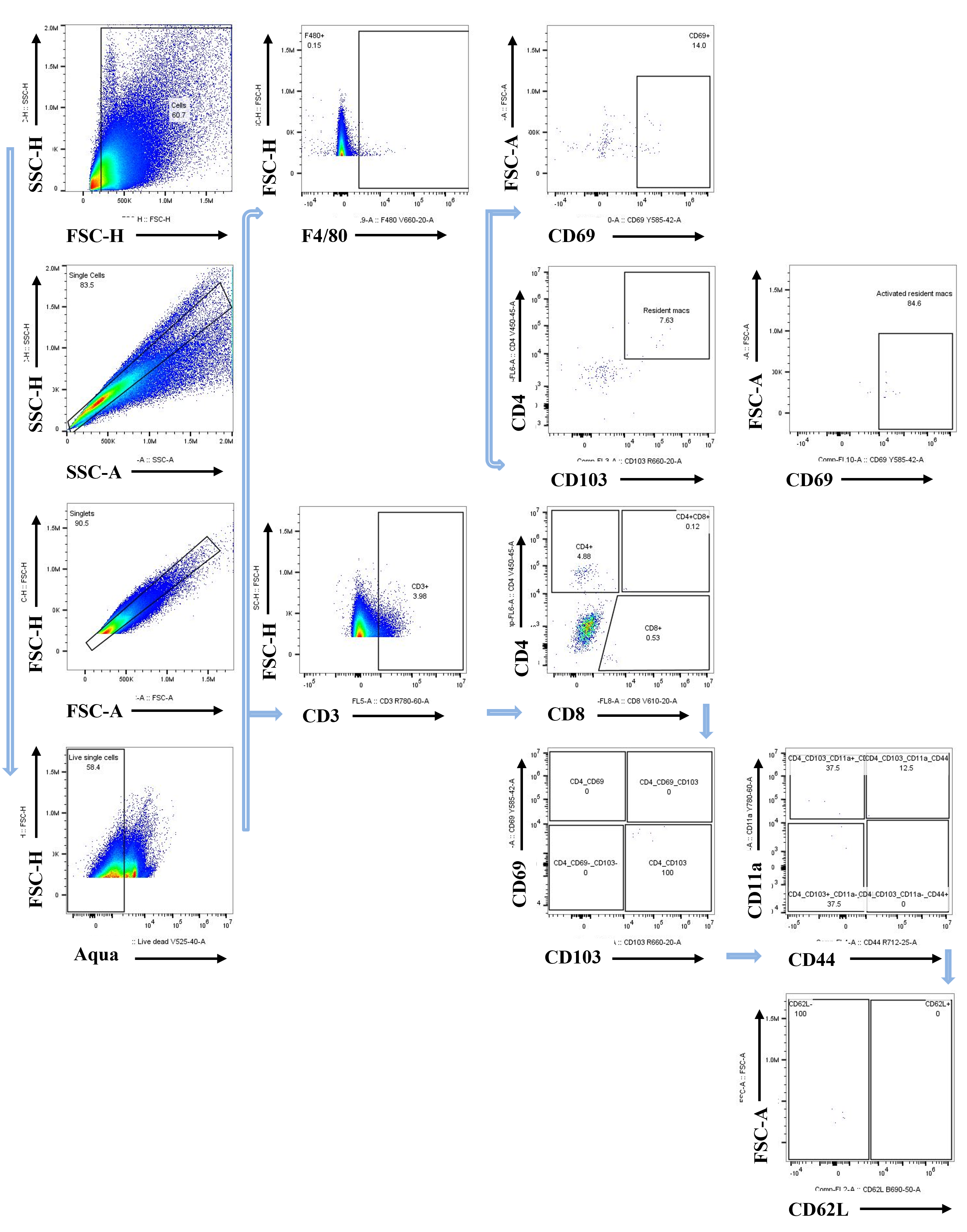

Supplement: Supplementary_Figure_5_ioad021 [file supplementary_figure_5_ioad021.zip › Supplementary_Figure_5_ioad021.tif]

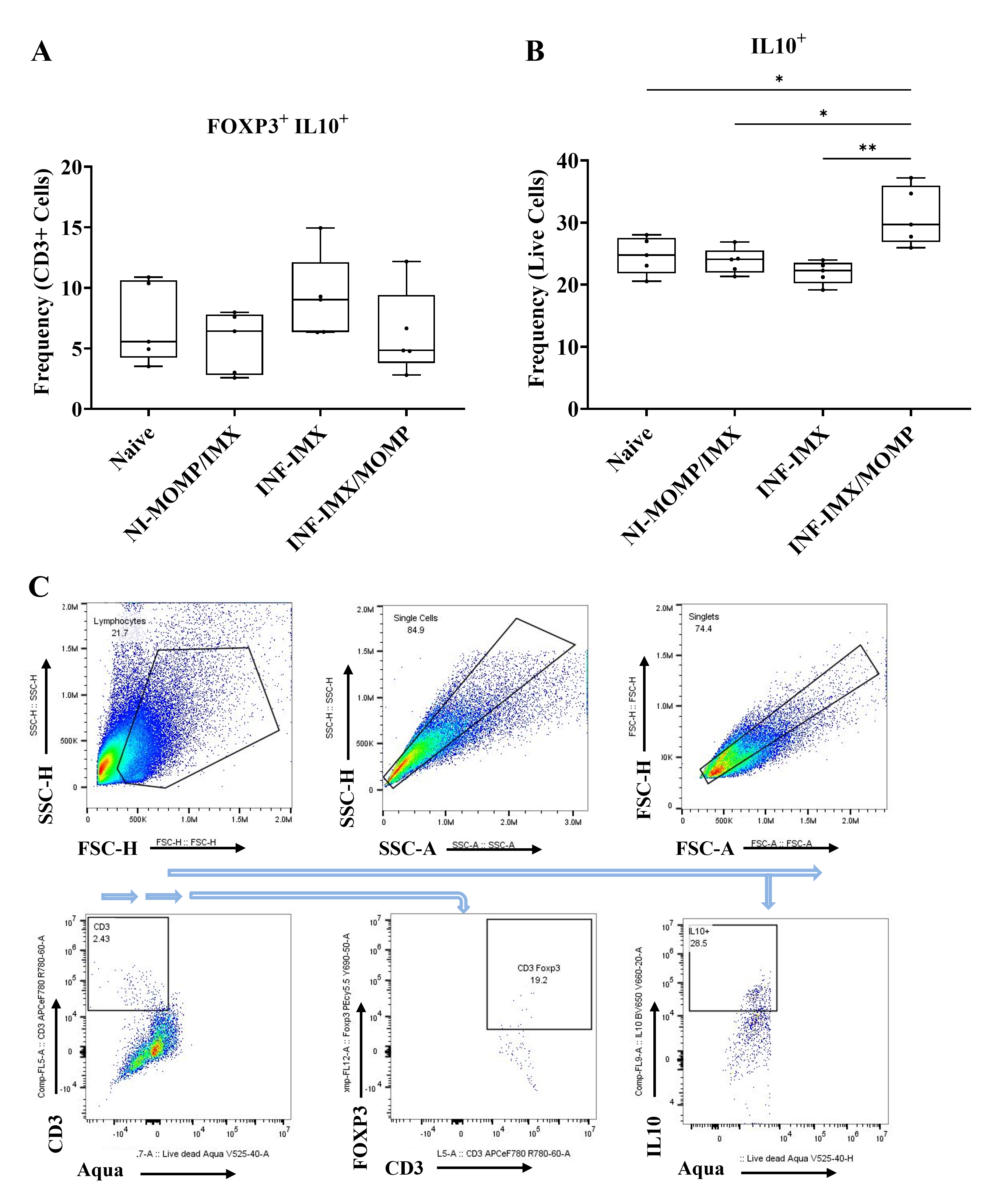

Supplement: Supplementary_Figure_6_ioad021 [file supplementary_figure_6_ioad021.zip › Supplementary_Figure_6_ioad021.tif]

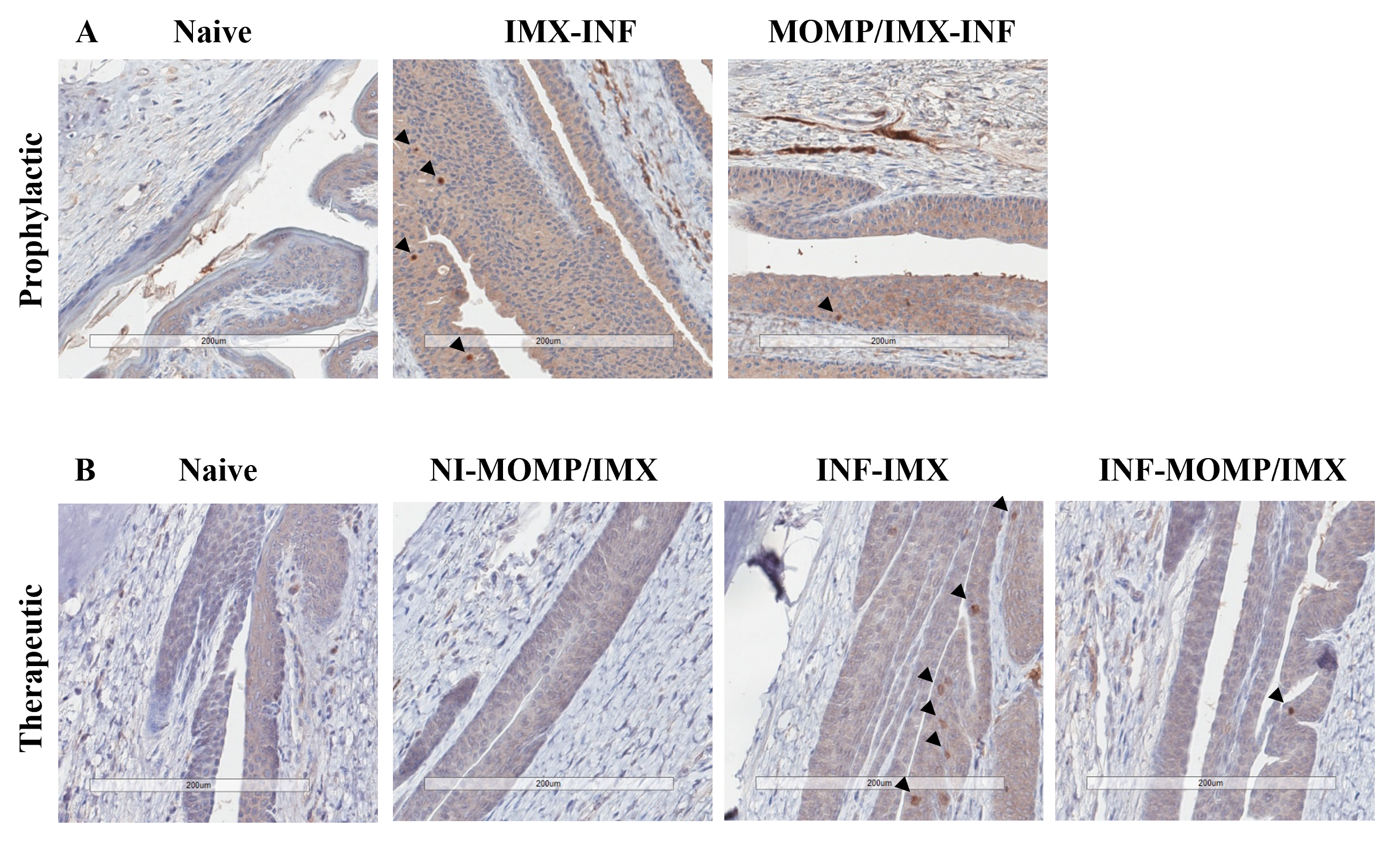

Supplement: Supplementary_Figure_7_ioad021 [file supplementary_figure_7_ioad021.zip › Supplementary_Figure_7_ioad021.tif]

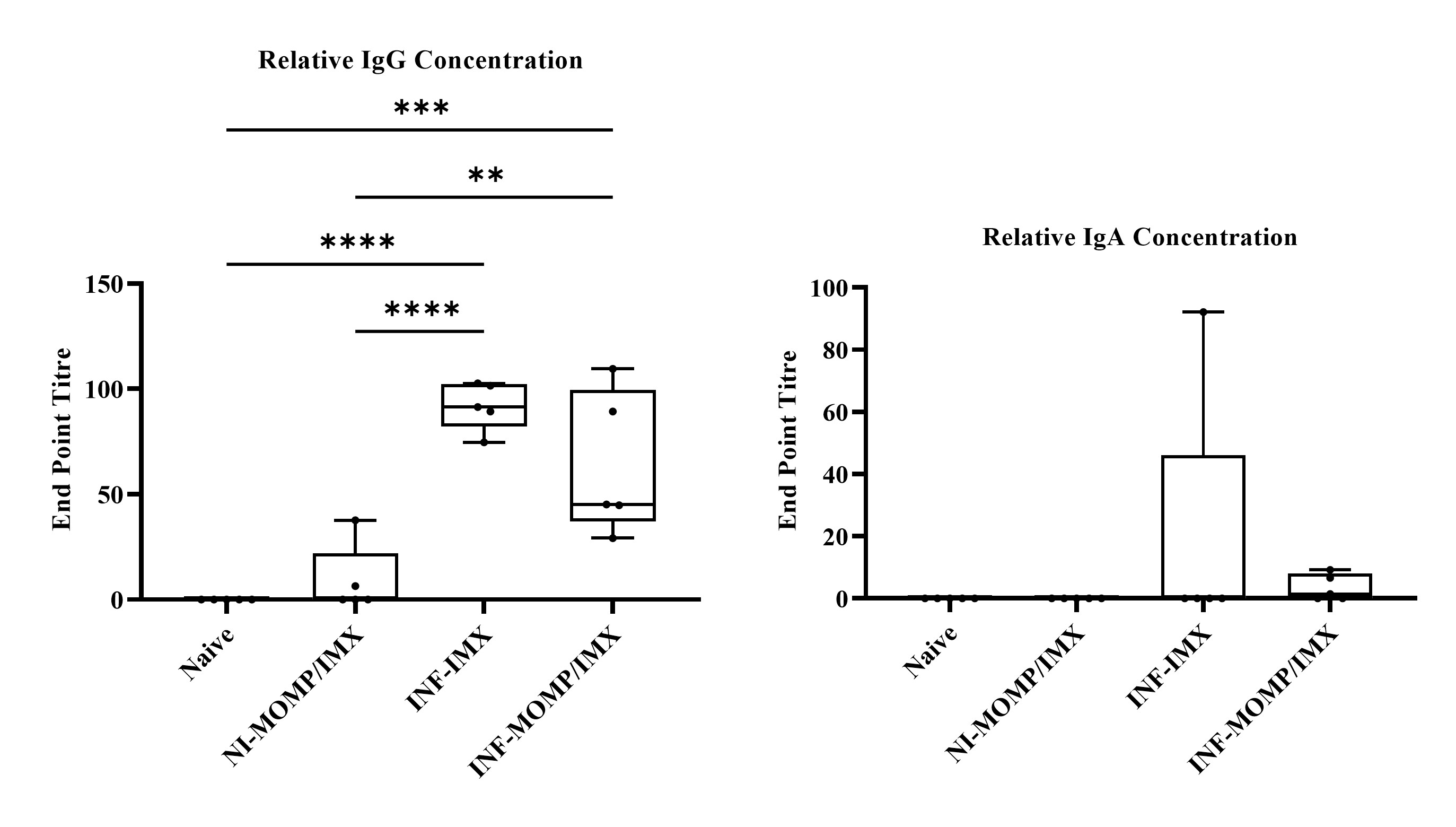

Supplement: Supplementary_Figure_8_ioad021 [file supplementary_figure_8_ioad021.jpeg]

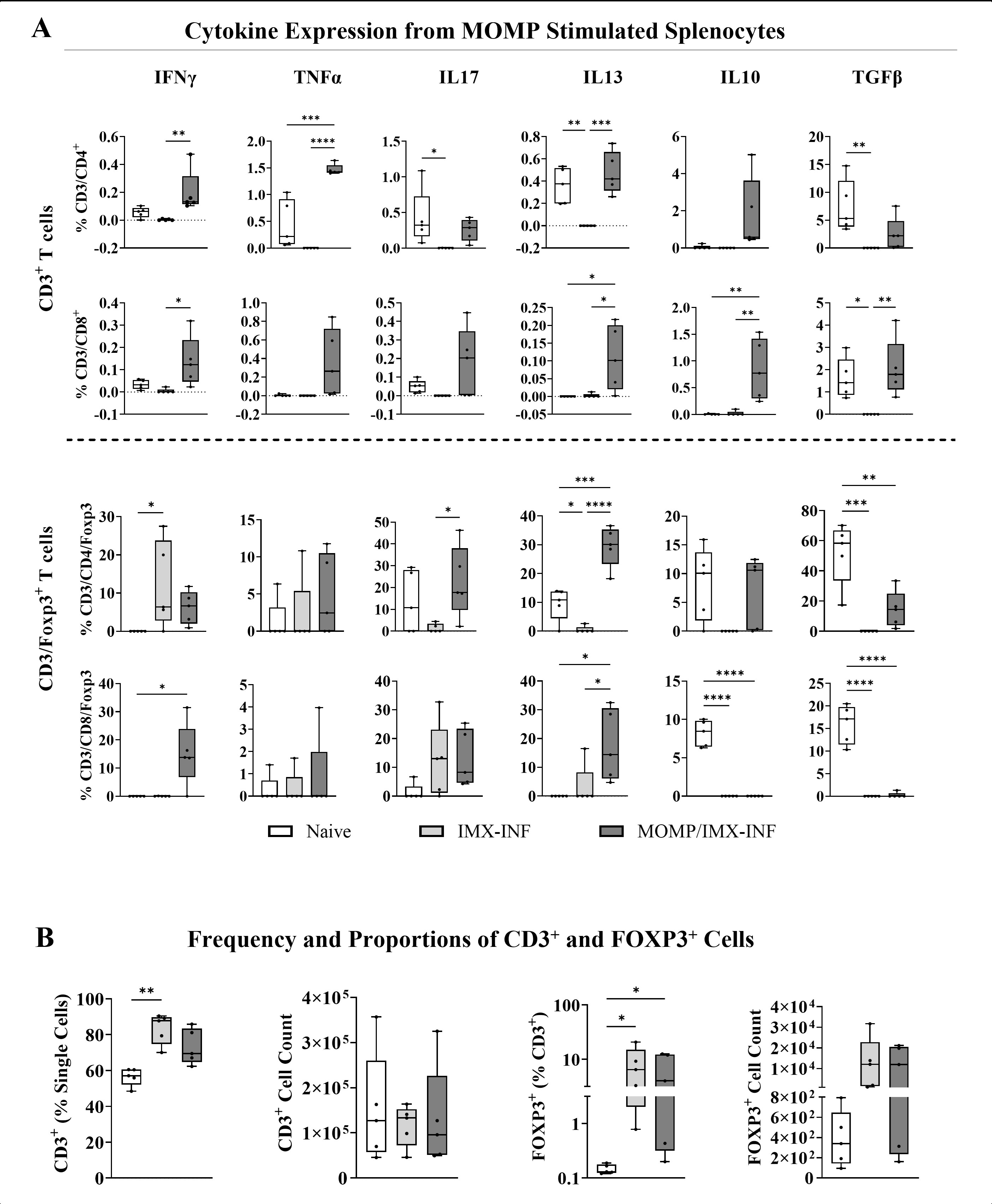

Supplement: Supplementary_Figure_9_ioad021 [file supplementary_figure_9_ioad021.jpeg]

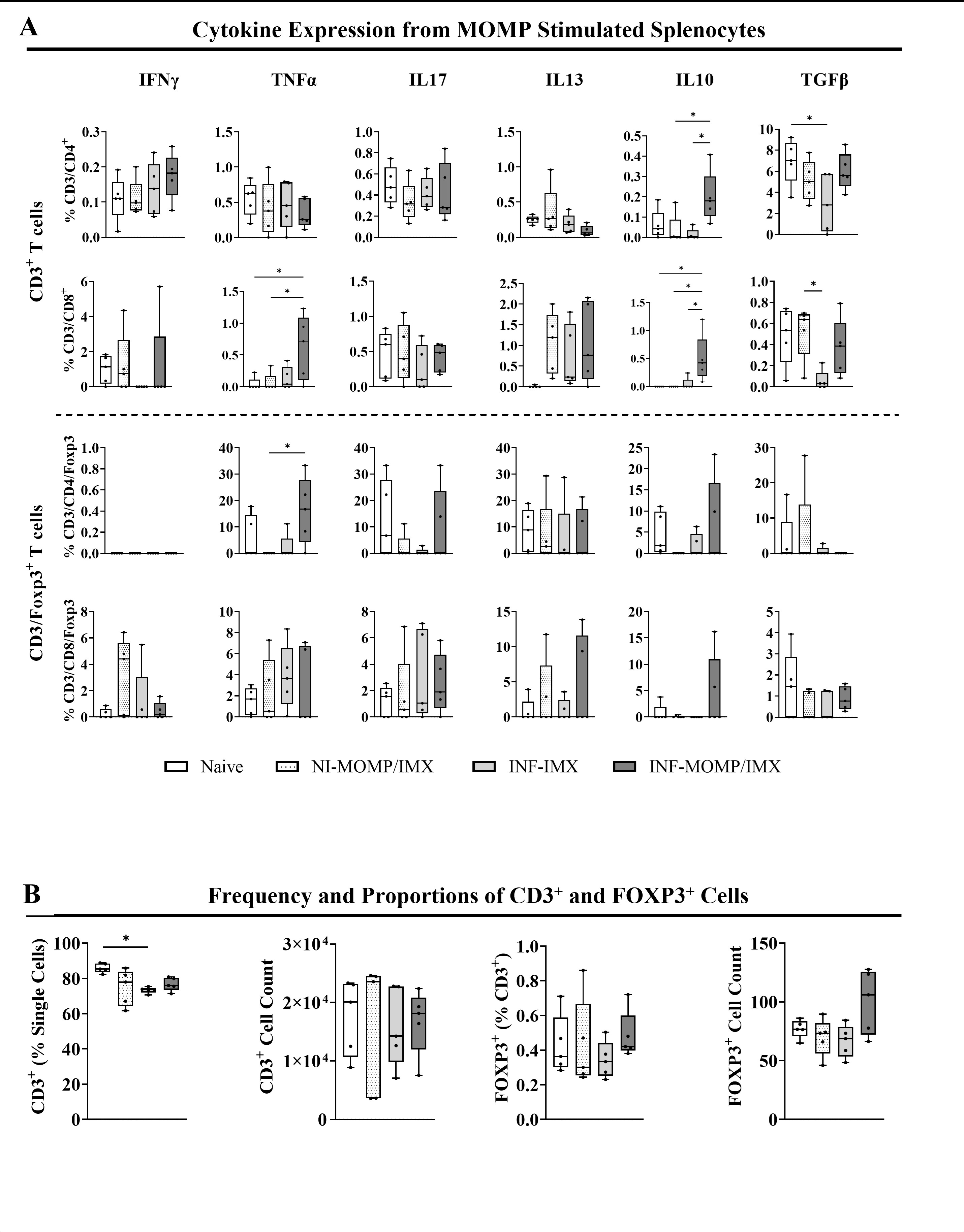

Supplement: Supplementary_Figure_10_ioad021 [file supplementary_figure_10_ioad021.jpeg]

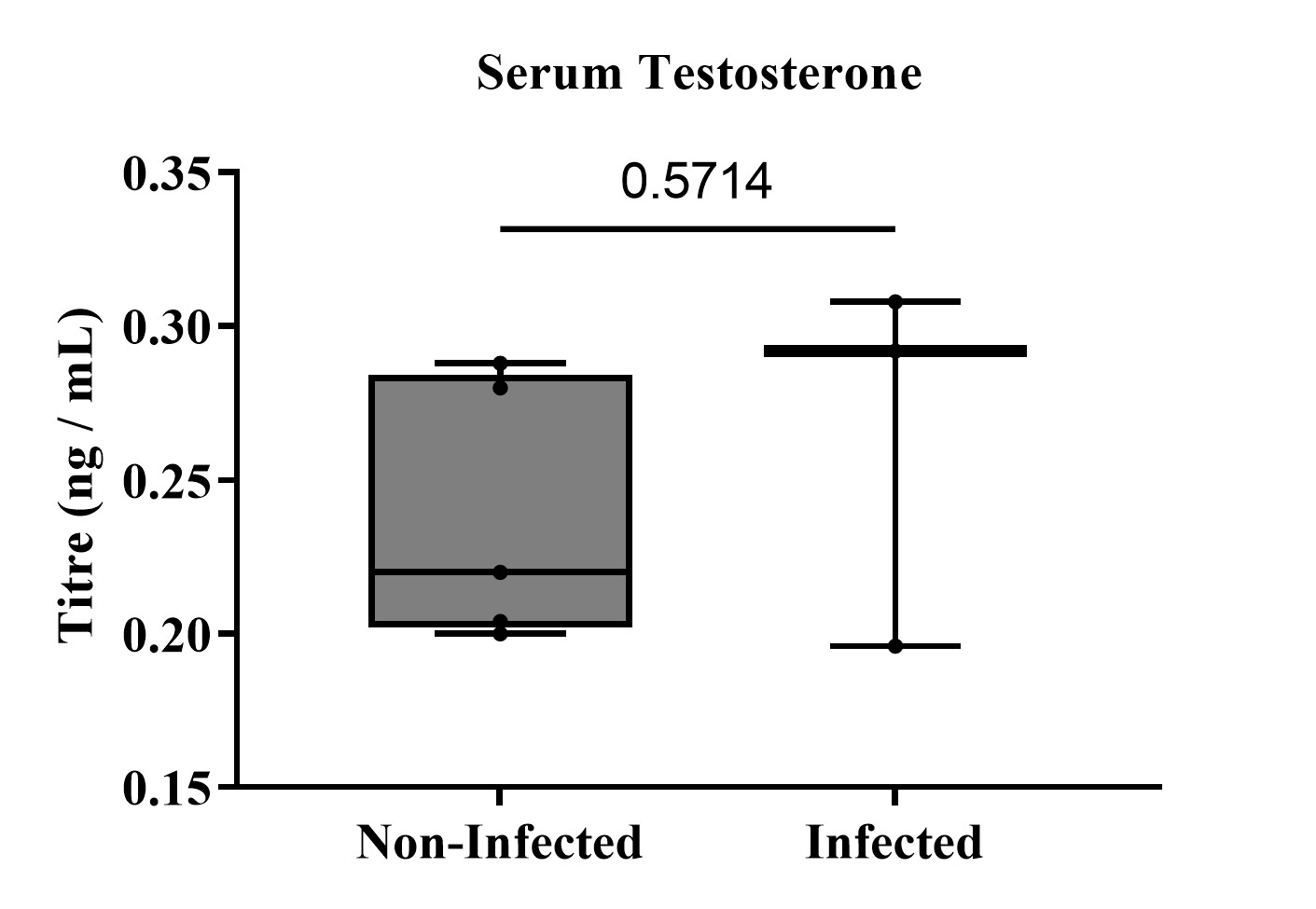

Supplement: Supplementary_Figure_11_ioad021 [file supplementary_figure_11_ioad021.jpeg]
