# Supplementary material for: Prophylactic and therapeutic vaccination protects sperm health from Chlamydia muridarum-induced abnormalities
Source: Biol Reprod. 2023 Feb 17;108(5):758–77. doi: 10.1093/biolre/ioad021 (PMC10183362; doi:10.1093/biolre/ioad021)
Supplement: Supplementary_Table_1_ioad021 [file supplementary_table_1_ioad021.docx]

Table 1: Flow cytometry antibody listings.

| **Marker** | **Product** | **Dilution** |
| --- | --- | --- |
| CD3e | Thermo Fisher Scientific, 47-0031-82 | 1:500 |
| CD4 | Thermo Fisher Scientific, 62-0042-82 | 1:500 |
| CD8a | Thermo Fisher Scientific, 63-0081-82 | 1:125 |
| IFNγ | Becton Dickson, 554413 | 1:250 |
| TNFα | Becton Dickson, 563944 | 1:1000 |
| TGFβ/LAP | Thermo Fisher Scientific, 25-9821-82 | 1:1000 |
| IL17A | Becton Dickson, 559502 | 1:250 |
| IL10 | Becton Dickson, 564083 | 1:500 |
| IL13 | Thermo Fisher Scientific, 61-7133-82 | 1:125 |
| FOXP3 | Thermo Fisher Scientific, 35-5773-82 | 1:500 |
| CD44 | Thermo Fisher Scientific, 56-0441-82 | 1:500 |
| CD62L | Thermo Fisher Scientific, 45-0621-82 | 1:500 |
| CD69 | Thermo Fisher Scientific, 12-0691-82 | 1:250 |
| CD11a | Thermo Fisher Scientific, 25-0111-82 | 1:1000 |
| CD103 | Thermo Fisher Scientific, 17-1031-82 | 1:500 |
| F4/80 | BioLegend, 123149 | 1:250 |
