# Supplementary material for: Prophylactic and therapeutic vaccination protects sperm health from Chlamydia muridarum-induced abnormalities
Source: Biol Reprod. 2023 Feb 17;108(5):758–77. doi: 10.1093/biolre/ioad021 (PMC10183362; doi:10.1093/biolre/ioad021)
Supplement: Supplementary_Table_2_ioad021 [file supplementary_table_2_ioad021.docx]

**Supplementary Table 2. Parameters for SWATH data analysis in Skyline software.**

| ***Peptide Settings*** | | |
| --- | --- | --- |
| Digestion | Enzyme | Trypsin [KR \| P] |
|  | Max missed cleavages | 1 |
|  | Background proteome | Generated from FASTA that was used for DDA data analysis |
|  | Enforce peptide uniqueness by | Proteins |
| Prediction | Retention time predictor | Created |
|  | Use measured retention time when present | Checked |
|  | Time window (min) | 3 |
|  | Ion mobility predictor | None |
|  | Use spectral library drift times when present | Unchecked |
|  | Resolving power | (blank) |
| Filter | Min length | 6 |
|  | Max length | 25 |
|  | Exclude N terminal AAs | 25 |
|  | Exclude potential ragged ends | Unchecked |
|  | Exclude peptides containing | (blank) |
|  | Auto select all matching peptides | Checked |
| Library | Library | Generated from DDA data |
|  | Pick peptides matching | Library and Filter |
|  | Rank peptides by | (blank) |
|  | Limit peptides per proteins | Unchecked |
|  | Peptides | (blank) |
| Modifications | Structural modifications | Carbamidomethyl (C) |
|  | Max variable mods | 3 |
|  | Max losses | 1 |
|  | Isotope label type | Heavy |
|  | Isotope modifications | (blank) |
|  | Internal standard type | None |
| Quantification | Normalization Method | Equalize Medians |
|  | All others | (blank) |
| ***Transition Settings*** | |  |
| Prediction | Precursor mass | Monoisotopic |
|  | Product ion mass | Monoisotopic |
|  | Collision Energy | None |
|  | Declustering potential | None |
|  | Optimization library | None |
|  | Compensation voltage | None |
|  | Use optimization values when present | Unchecked |
| Filter | Precursor charges | 2, 3, 4 |
|  | Ion charges | 1, 2, 3 |
|  | Ion types | y, b |
|  | Product ion selection From | Ion 3 |
|  | To | Last ion |
|  | Special ions | N-terminal to Proline |
|  | Use DIA precursor window for exclusion | Checked |
|  | Auto select all matching transitions | Checked |
| Library | Ion match tolerance (*m/z*) | 0.05 |
|  | If a library spectrum is available, pick its most intense ions | Checked |
|  | Pick product ions | 5 |
|  | Minimum product ions | 5 |
|  | From filtered ions charges and types | Unchecked |
|  | From filtered ions charges and types plus filtered product ions | Unchecked |
|  | From filtered product ions | Checked |
| Instrument | Min *m/z* | 400 |
|  | Max *m/z* | 1,500 |
|  | Dynamic min product *m/z* | Unchecked |
|  | Method match tolerance *m/z* | 0.055 |
|  | Firmware transition limit | (blank) |
|  | Firmware inclusion limit | (blank) |
|  | Min time (min) | (blank) |
|  | Max time (min) | (blank) |
|  | Triggered chromatogram acquisition | Unchecked |
| Full-Scan | MS1 filtering - Isotope peaks included | None |
|  | Precursor mass analyser | (blank) |
|  | Peaks | (blank) |
|  | Resolution (*m/z*) | (blank) |
|  | Isotope labelling enrichment | (blank) |
|  | MS/MS filtering - Acquisition method | DIA |
|  | Product mass analyser | TOF |
|  | Isolation scheme | Generated from SWATH data file as follows:  399.50, 411.00, 5  410.00, 421.20, 5  420.20, 429.90, 5  428.90, 438.30, 5  437.30, 446.00, 5  445.00, 453.00, 5  452.00, 459.70, 5  458.70, 466.70, 5  465.70, 473.30, 5  472.30, 479.60, 5  478.60, 485.60, 5  484.60, 491.50, 5  490.50, 497.50, 5  496.50, 503.80, 5  502.80, 509.70, 5  508.70, 516.00, 5  515.00, 522.00, 5  521.00, 527.90, 5  526.90, 534.20, 5  533.20, 540.90, 5  539.90, 547.50, 5  546.50, 554.50, 5  553.50, 561.20, 5  560.20, 568.20, 5  567.20, 574.80, 5  573.80, 581.80, 5  580.80, 588.80, 5  587.80, 595.80, 5  594.80, 602.80, 5  601.80, 610.20, 5  609.20, 617.90, 5  616.90, 625.20, 5  624.20, 632.60, 5  631.60, 639.90, 5  638.90, 647.30, 5  646.30, 655.00, 5  654.00, 662.70, 5  661.70, 671.10, 5  670.10, 679.80, 5  678.80, 689.30, 5  688.30, 699.10, 5  698.10, 708.90, 5  707.90, 718.30, 5  717.30, 728.10, 5  727.10, 738.30, 5  737.30, 748.80, 5  747.80, 759.60, 5  758.60, 770.80, 5  769.80, 782.70, 5  781.70, 795.30, 5  794.30, 809.00, 5  808.00, 823.70, 5  822.70, 839.40, 5  838.40, 855.90, 5  854.90, 873.00, 5  872.00, 890.90, 5  889.90, 911.90, 5  910.90, 937.10, 5  936.10, 967.20, 5  966.20, 1000.00, 5 |
|  | Resolving power | 30,000 |
|  | Use high-selectivity extraction | Unchecked |
|  | Retention time filtering - Use only scans within minutes of MS/MS IDs | Unchecked |
|  | Use only scans within minutes of predicted RT | 8 |
|  | Include all matching scans | Unchecked |
| Ion Mobility | Ion mobility filtering – Ion mobility library | None |
|  | Use spectral library ion mobility values when present | Unchecked |
|  | Window type | None |
| ***Document Settings*** | | |
| Annotations | | Condition  BioReplicate |
| Result Files | | (blank) |
| Lists | | (blank) |
| Group Comparisons | | Control vs MOMP  Control vs IMX  IMX vs MOMP |
| Reports | | Unchecked |
| ***Integrate All*** | | Checked |
| ***Reintegrate*** | | |
| Peak Scoring Model – Choose model | | mProphet |
| Training - Use Decoys | | Checked |
| Training - Use second best peaks | | Unchecked |
| Integrate all peaks | | Checked |
| Only integrate significant q values | | Checked |
| Q value cutoff | | 0.01 |
| Overwrite manual integration | | Checked |
